# Nicotine in Inflammatory Diseases: Anti-Inflammatory and Pro-Inflammatory Effects

**DOI:** 10.3389/fimmu.2022.826889

**Published:** 2022-02-18

**Authors:** Wenji Zhang, Hui Lin, Mingmin Zou, Qinghua Yuan, Zhenrui Huang, Xiaoying Pan, Wenjuan Zhang

**Affiliations:** ^1^ Guangdong Provincial Engineering & Technology Research Center for Tobacco Breeding and Comprehensive Utilization, Key Laboratory of Crop Genetic Improvement of Guangdong Province, Crops Research Institute, Guangdong Academy of Agricultural Sciences, Guangzhou, China; ^2^ Department of Radiation Oncology, Guangdong Provincial People’s Hospital, Guangdong Academy of Medical Sciences, Guangzhou, China; ^3^ Department of Public Health and Preventive Medicine, School of Medicine, Jinan University, Guangzhou, China

**Keywords:** nicotine, anti-inflammation, pro-inflammation, nAChRs, immune, vagus

## Abstract

As an anti-inflammatory alkaloid, nicotine plays dual roles in treating diseases. Here we reviewed the anti-inflammatory and pro-inflammatory effects of nicotine on inflammatory diseases, including inflammatory bowel disease, arthritis, multiple sclerosis, sepsis, endotoxemia, myocarditis, oral/skin/muscle inflammation, *etc.*, mainly concerning the administration methods, different models, therapeutic concentration and duration, and relevant organs and tissues. According to the data analysis from recent studies in the past 20 years, nicotine exerts much more anti-inflammatory effects than pro-inflammatory ones, especially in ulcerative colitis, arthritis, sepsis, and endotoxemia. On the other hand, in oral inflammation, nicotine promotes and aggravates some diseases such as periodontitis and gingivitis, especially when there are harmful microorganisms in the oral cavity. We also carefully analyzed the nicotine dosage to determine its safe and effective range. Furthermore, we summarized the molecular mechanism of nicotine in these inflammatory diseases through regulating immune cells, immune factors, and the vagus and acetylcholinergic anti-inflammatory pathways. By balancing the “beneficial” and “harmful” effects of nicotine, it is meaningful to explore the effective medical value of nicotine and open up new horizons for remedying acute and chronic inflammation in humans.

## 1 Introduction: Nicotine in the Tobacco Plant

Nicotine, also called 3-(1-methyl-2-pyrrolidinyl) pyridine, normally accounts for about 5% (w/w) of the tobacco plant and is the major psychoactive and addictive component in tobacco smoke. It belongs to alkaloids, with a pyridine cycle and a pyrrolidine cycle, and occupies a higher proportion of ~95% of the total alkaloids in tobacco leaves (1). Nicotine is often blamed for its associations with smoking and addiction. However, with a further clinical understanding of nicotine in neurodegenerative diseases such as Alzheimer’s and Parkinson’s diseases due to its positive anti-inflammatory, anti-apoptotic, pro-cognitive, and anti-protein aggregation effects (2, 3), its medical value has attracted considerable attention.

Undoubtedly, smoking increases the risk of many diseases, such as cancer, coronary heart disease, stroke, and chronic obstructive pulmonary disease. However, tobacco was first found because of its valuable and pharmacological effects in history (4, 5). As an herbal medicine, its medicinal benefits, including treating ulcerated abscesses, fistulas, sores, inveterate polyps, headaches, and many other ailments, were tremendous before the 19th century in Europe and especially in South America (6). Concerning the tobacco plant, in addition to the controversial nicotine, it also contains a considerable amount of solanesol, chlorogenic acid, rutin, and other widely recognized beneficial ingredients that have antioxidant, anti-inflammatory, anti-microbial, anti-obesity, anti-pyretic, neuroprotective, and anti-hypertensive activities and so on (7–9). Therefore, we agree with Anne Charlton that we should set aside the prejudices generated by the ill effects of tobacco smoking, sensibly distinguish the tobacco plant, tobacco smoking, and the included substances of therapeutic value, and call for further research (6).

A quote has been bouncing around the tobacco research community since the 1970s that “people smoke for the nicotine, but they die from the tar.” It might be difficult to differentiate the effects of nicotine from many other toxic substances (10). The studies on lung toxicity of e-cigarette also showed that nicotine itself had almost no influence on the modulation of the toxicity response or lipid homeostasis in alveolar macrophages and epithelial cells, while flavor composition or vehicle solvents did have (11, 12). Here we focused on the medical action about monomer nicotine only; we should neither avoid its toxic effects nor ignore its pharmacological effects. Importantly, we hope that the review will elucidate the positive role of nicotine in human diseases in the future.

## 2 Nicotine and Inflammation

Inflammation underlies various physiological and pathological processes, including acute inflammation in infection, trauma, surgery, burns, ischemia, or necrotic tissue and chronic inflammation in allergy, atherosclerosis, cancer, arthritis, autoimmune diseases, *etc.* (13, 14). Inflammation is a complex process involving multiple genes and signaling pathways. In the recent decade, the finding that pro-inflammatory responses are controlled by neural circuits has given birth to the new concept of “inflammatory reflex.” The cholinergic anti-inflammatory pathway is the efferent or motor arm of the “inflammatory reflex”, the neural circuit that responds to and regulates the inflammatory response (15). In the neural circuit, the neurotransmitter endogenic acetylcholine, for example, can interact with the receptors expressed on immune cells and thus alter immune cell function (16). It is generally acknowledged that acetylcholine can bind to the integral membrane protein as acetylcholine receptors, including nicotinic acetylcholine receptors (nAChR) and muscarinic acetylcholine receptors (mAChR). It is well known that nicotine, as an agonist of nAChR found in the central and peripheral nervous system, muscle, and many other tissues of many organisms (17), stimulates the nicotinic acetylcholine receptor signaling anti-inflammatory pathway to reduce inflammatory responses, depression, attention deficit–hyperactivity disorder, cognitive deficits in schizophrenia, Alzheimer’s disease, and pain (18). Furthermore, nicotine is a lipophilic agent and can penetrate the cells independently on these special nAChR receptors (19). Therefore, nicotine could directly affect mitochondrial respiration, cell autophagy, and cell signaling molecules in an environment with proper pH (nicotine pKa = 7.9) (20). In our review, we found that nicotine acts through many signaling pathways in addition to the mainstream cholinergic anti-inflammatory pathway and nAChRs.

What makes people more concerned is the inhibitory effect of nicotine on cytokine storm in lungs in severe respiratory symptoms in the recent epidemic era of COVID-19. People tend to support a potential therapeutic role for nicotine in COVID-19, owing to the varied effects, including mood regulation, anti-inflammatory effects, and purported interference with SARS-CoV-2 entry and/or replication (21, 22). Of course, the effect of nicotine on inflammation is not limited to these aspects. In our study, we found many uncertain factors that affect the function of nicotine or even reverse it.

In order to clarify the immunomodulatory regulation of nicotine, we analyzed its function in human inflammatory diseases. A comprehensive literature search from the year 2000 was conducted through PUBMED using the search terms “anti-inflammatory and nicotine”, “anti-inflammation and nicotine”, “pro-inflammatory and nicotine”, “inflammation and nicotine”, and “pro-inflammation and nicotine”. Relevant articles on human inflammatory diseases were identified carefully through the manual review. Concerning the divided voices on the tobacco plant and modern medical research, we hoped to evaluate the medicinal value of nicotine and avoid the toxic effects cautiously, eventually making it a positive choice for human health in medicine.

## 3 Nicotine-Related Inflammatory Diseases

**Figure 1:**
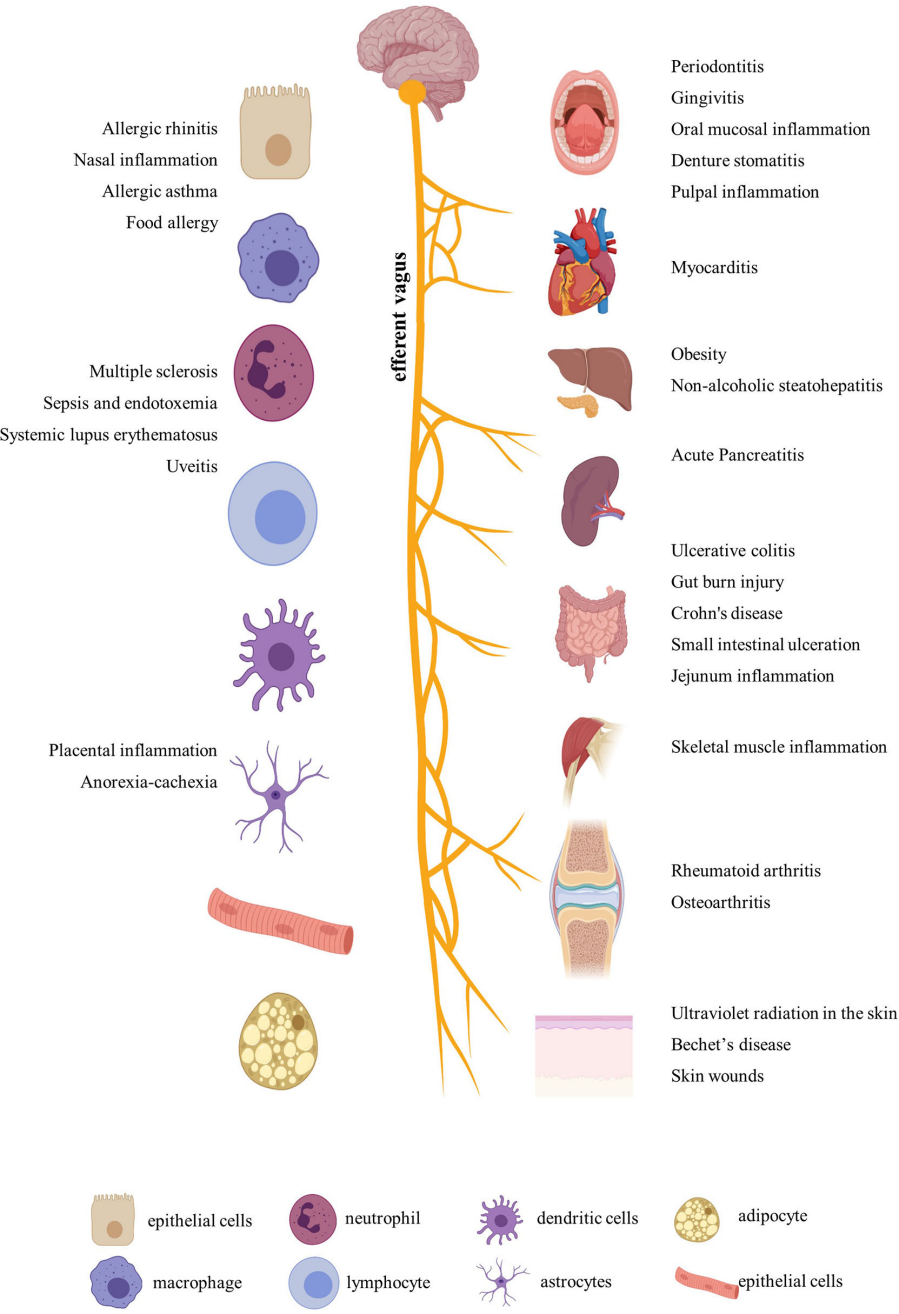
Inflammatory diseases and related organs and cells regulated by nicotine. Created with BioRender.

After a comprehensive review of the published papers, we figured out that nicotine participated in more than 20 diseases by immune regulation. In addition to the most-studied neurodegenerative diseases, including Parkinson’s disease and Alzheimer’s disease (23), nicotine also played different regulatory roles in ulcerative colitis, arthritis, periodontitis, sepsis, endotoxemia, multiple sclerosis, nasal eosinophilic inflammation, allergy, nonalcoholic fatty liver disease, nonalcoholic steatohepatitis, skin inflammation, placental inflammation, pancreatitis, Behçet’s disease, muscle inflammation, viral myocarditis, uveitis, experimental autoimmune encephalomyelitis, systemic lupus erythematosus, and so on (**Figure 1**). In addition, for easy inquiry, summarized and listed in **Supplementary Table S1** are all the reviewed diseases, cell and animal models, nicotine dosage, administration methods, and target factors, followed by evaluation and comparison on the effect efficacy.

Based on former research, we updated the role of nicotine in the following inflammatory diseases in this part. No matter in a common or a rare disease, nicotine may have significant effects on alleviating human diseases in the future.

### 3.1 Inflammatory Bowel Disease

Inflammatory bowel disease (IBD) is a global problem. It impacts all aspects of life and affects people of all ages, most commonly during adolescence and young adulthood (24). It usually includes two major distinct forms: ulcerative colitis and Crohn’s disease. Clinical analyses have shown that smoking worsens Crohn’s disease but is beneficial for ulcerative colitis for many years due to nicotine and possibly flavonoid components in the smoke extract (25, 26). However, nicotine monomers have a dual effect on these two diseases. This chapter also discusses inflammatory bowel disease, including burn-induced histological gut injury and small intestine ulceration.

#### 3.1.1 Anti-Inflammatory Effect of Nicotine on IBD

In general, nicotine is beneficial in ulcerative colitis; in particular, nicotine transdermal patches or nicotine enemas have shown significantly improved histological and global clinical scores of colitis, inhibited pro-inflammatory cytokines in macrophages, and induced protective autophagy to maintain intestinal barrier integrity (27, 28). In ulcerative colitis patients, 6 mg of both oral and transdermal nicotine could be well tolerated, and this is thus thought to be the highest therapeutic dose with a low risk of adverse effects in the human body (29). However, nicotine’s unspecific effects limit its clinical applications. Even so, the exploration of its therapeutic effect on ulcerative colitis has never stopped. Scientists hope to determine its roles through comprehensive reviews and analyses.

The most commonly used animal model of ulcerative colitis is the dextran sodium sulfate (DSS)-induced mouse or rat model, with body weight loss, colon shortening, missing colonic mucosal epithelial cell, gland integrity loss, inflammatory cell infiltration, colonic tissue injury, and typical inflammatory reaction. In DSS-induced male C57BL/6J mice, nicotine (0.1 mg/ml) administration *via* the drinking water suppressed the mucosal vascular address in cell adhesion molecule‐1 (MAdCAM‐1) expression on the endothelium of colonic microvessels and endothelial bEnd.3 cells and the recruitment of leukocytes to the inflamed colonic microvessels, which is the underlying pathophysiology of DSS‐induced colitis (30). Oral nicotine (6, 12.5, 25, and 50 μg/ml, not 100 μg/ml, in drinking water), subcutaneous nicotine (0.1 mg/kg), and minipump infusion (2.5 mg/kg) attenuated the DSS-induced colitis of male C57BL/6 mice, which resulted in a significant decrease in histologic damage scores with myeloperoxidase (MPO) and TNFα reduction. However, subcutaneous nicotine (0.5 and 2 mg/kg) and minipump infusion (25 mg/kg) treatment failed to significantly alter the DSS-treated damage (31). Nicotine (10 µg/kg/day, gavage) significantly reduced the histologic inflammation in colon tissues by reducing the levels of TNFα, IL-1β, and IL-6 and cell apoptosis and induced autophagy *via* the AMPK–mTOR–P70S6K pathway in DSS-induced adult male C57BL/6 mice to treat colitis, along with enhanced LC3II/LC3I and beclin-1 and decreased p62 protein level (32). Furthermore, by improving IL-1β, subcutaneous nicotine administration (2 mg/kg, b.i.d.) suppressed the hyperexcitability of colonic dorsal root ganglia (L_1_–L_2_) neurons and the visceral hypersensitivity of the number of abdominal constrictions in response to an intraperitoneal injection of 0.6% acetic acid in DSS-treated male C57BL/6J mice, finally relieving acute DSS colitis (33). In female C57BL/6 mice, nicotine (0.25 and 2.50 μmol/kg, i.p.) treatment reduced the activation of NF-κB and colonic cytokine production, including IL-6, IL-17, and TNF, but failed to reduce the disease parameters in DSS colitis. However, it failed in 2,4,6-trinitrobenzene sulphonic acid (TNBS)-induced colitis (34). It seems that nicotine may work better in male C57BL mice than in female ones. In other DSS-induced mice and in male CD1 mice, oral treatments with nicotine (0.1, 0.3, and 1.0 mg/kg, p.o.) were effective in inhibiting the mechanical hyperalgesia caused by colonic inflammation in mice *via* α7 nAchR activation and IL-1β suppression, with no effect on DSS-induced colonic damage or inflammation in 7 days (35). In male BALB/C mice, Hayashi et al. (36) also found that nicotine (3 mg/kg, s.c.) significantly attenuated the severity of DSS-induced colitis by decreasing the MPO activity, suppressing IL-6 mRNA, and activating the α7-nAChR receptor of colon and isolated CD4 T cells. Furthermore, they proved that nicotine significantly reduced the number and size of colonic tumors in mice with colitis-associated cancer induced by administering azoxymethane and DSS through the IL-6/Stat3/miR-21 signaling pathway. Thus, nicotine could suppress DSS-induced acute colitis and also colonic tumorigenesis associated with chronic colitis in mice. The most direct evidence showed that the nicotinic acetylcholine receptor played important mediating roles in DSS-induced ulcerative colitis using nAChRs-deficient mice. Alpha 5 nicotinic acetylcholine receptor subunits played key roles in this disease. In the DSS-induced mouse model of acute colonic inflammation, injury in a5^-/-^ mice involved the entire bowel wall thickness, with fissuring ulcers and moderate inflammatory infiltrates in the mucosa, submucosa, and the inner muscular layer. Nicotine (12.5 mg/ml) in the drinking water of a5-deficient mice for 7 days before the induction still significantly ameliorated the colonic inflammation course by reducing prostaglandin E2 (PGE2) and MPO, indicating that other nicotinic acetylcholine receptor subunits also mediate the anti-inflammatory effects of nicotine (37). α7 nAchR was shown to be necessary and dependent when nicotine suppressed the hyperexcitability of colonic dorsal root ganglia (L_1_–L_2_) neurons *in vitro* (28) and *in vivo* (bath-applied) (33). Neuronal nAChR in anti-inflammatory mechanisms is a newer field of investigation in colonic inflammation. These findings complement the anti-inflammatory effects of nicotine, which work together to treat colitis.

In other models of different inducers in different animals, nicotine played effective roles in tolerating dosages, illustrating the wide range of nicotine use in colitis. In hapten (2,4-dinitrobenzene sulfonic acid)-induced colitis in Sprague–Dawley rats, subcutaneous nicotine (a single dose of 8 mg/kg or triple doses of 4 mg/kg nicotine) reduced the colonic lesion size, MPO activity, luminol-amplified free radical generation, and leukotriene B4 (LTB_4_) formation by downregulating the colonic IL-1β and monocyte chemoattractant protein (MCP)-1 protein expression in the inflamed colon of animals with colitis (25). In acetic acid-induced colitis of Sprague–Dawley rats, nicotine (1 mg/kg, i.p.) reduced the extent of colonic lesions, colonic malondialdehyde (MDA) level, MPO activity, NF-κB expression, and serum IL-1β level. Also, nicotine reversed the decreased serum visfatin levels in the colitis group (38). In *Clostridium difficile* toxin A-induced colitis in male Sprague–Dawley rats, 30 min of pretreatment with nicotine (2 ng, 20 ng, 200 ng, 2 μg, and 20 μg) in the isolated colonic segments induced by toxin A dose-dependently inhibited the harmful effects, but not the ileum experiment. Furthermore, nicotinic inhibition includes the downregulation of transient receptor potential vanilloid subtype 1, LTB_4_, and pro-inflammatory neuropeptide (substance P) *via* nAChRs, but not directly (39). In the oxazolone-induced colitis of female BALB/C mice, nicotine (2.5 mg/kg, s.c.) attenuated inflammation by increasing the proportion of CD25^+^Foxp3^+^ Tregs and decreasing CD4^+^IL-17^+^ Tregs in colonic CD4-positive cells (40).

Except colitis, in other diseases, such as burn-induced histological gut injury, male BALB/C mice were given nicotine (400 μg/kg, i.p.) 30 min after a severe burn injury, which improved the gut barrier function of intestinal permeability by preventing the decreased expression of occludin and ZO-1 and maintaining their localization. In addition, nicotine was further shown to improve intestinal inflammation by inhibiting the stimulation of IFN-γ, TNF-α, and IL-1β in intestinal epithelial cell CaCO-2 cocultured with enteric glial cells (41). However, a similar effect was not found in the triculture intestinal model consisting of a differentiated intestinal epithelial layer (CaCO-2/HT29-MTX) and immunocompetent cells (differentiated THP-1) (42).

In small intestinal ulceration induced by indomethacin in male C57BL/6 mice, nicotine (0.3, 1, and 3 mg/kg, i.p. twice, 0.5 h before and 8 h after indomethacin treatment) reduced the severity of intestinal lesions by up to 53.3, 74.7, and 81.3%, respectively, in a dose-dependent manner by suppressing the MPO activity and iNOS expression due to the inhibition of NFκB. This effect was impaired by methyllycaconitine, a selective antagonist of α7 nAChR antagonist (43).

Regulation of inflammation in intestinal mesothelial cells in the abdominal cavity is essential for postoperative ileus pathogenicity. Nicotine (10 nM) significantly inhibited the lipopolysaccharide (LPS)-induced mRNA expression of IL-1β and iNOS, but not TNF-α and MCP-1, in primary male Sprague–Dawley rats’ mesothelial cells *via* their connectivity with enteric nerves expressing α7 nAChRs (44).

Although smoking is associated with increased incidence and severity of Crohn’s disease, nicotine exerts beneficial functions in different conditions. In the TNBS model of male Wistar rats’ model of Crohn’s disease, oral administration of nicotine (5 μg/ml, 10-day pretreatment or 3 days after induction, in drinking water) reduced macroscopic damage with a reduction in MPO, iNOS, TNFα, and LTB_4_. This effect was similar to 375 μg/ml sulphasalazine but greater than 100 μg/ml nicotine, indicating that low doses of nicotine were more effective in reducing inflammation than higher doses (45). In Crohn’s disease, nucleotide-binding oligomerization domain (NOD) 2 (NOD2), the first identified susceptibility gene, is also reduced by nicotine in HT29 cells when treated by TNF-α, implying the potential anti-inflammatory effect of nicotine on Crohn’s disease (46).

In a human experiment, in 30 patients with left-sided ulcerative colitis refractory to rectal mesalamine alone, by calculating the Rachmilewitzis activity index confirmed by endoscopic and histological findings, transdermal nicotine (15 mg) plus mesalamine enemas (4 g) showed significantly therapeutic success in 4 weeks than mesalamine tablets (oral mesalamine 800 mg t.i.d.) plus mesalamine enemas (4 g) (47).

Postoperative ileus, a transient cessation of bowel motility after surgery, was also improved by nicotine. Furthermore, in patients, nicotine gum reduced the time of postoperative ileus and improved patient outcomes *via* vagus nerve activation, with no apparent adverse events (48). Nicotine suppressed the release of inflammatory mediators and cytokines, including TNF-α, IL-1β, IL-6, IL-17, and IFN-γ, alleviated oxidative stress by decreasing luminol-amplified free radical generation, MPO, NO, and iNOS, inhibited immunocytic LTB4 formation and MCP-1 expression, and improved gut structural integrity by maintaining MAdCAM‐1, occludin, and ZO-1 by regulating the AMPK/mTOR/NF-κB/Stat3 signaling pathway and related factors such as miR-21. Nicotine also induced autophagy by enhancing LC3II/LC3I and beclin-1 and decreasing the p62 protein level. However, there are still some peculiarities involving nicotine’s selectivity in regulating immune cytokines and organs—for example, in interleukin-10-deficient male C57/BL10 mice, nicotine (12.5 μg/ml) for 2 weeks in their drinking water at the age of 12–14 weeks, when they had developed clinical signs of inflammatory bowel disease, significantly reduced the colonic scores but aggravated jejunum inflammation, possibly because nicotine significantly increased both somatostatin and intestinal trefoil factor mRNA expression in the colon but not in the jejunum (49).

#### 3.1.2 Pro-Inflammatory Effect of Nicotine on Inflammatory Bowel Disease

It can be seen from the discussion above that nicotine has much more positive effects on inflammatory bowel disease. There are also counterexamples. In TNBS-induced BALB/C mice model of Crohn’s disease, nicotine taken twice a day at a total dose of 7.5 mg/kg for 5 days exacerbated colonic inflammation with the appearance of extensive mucosal necrosis in the proximal colon, extensive crypt damage, complete goblet cell depletion, lamina propria destruction, and massive lymphocytic infiltration, which was associated with the inhibition of colonic CD4 T cell nAChRs regulated by IL-12 (40). Nicotine (4 µg/ml) modulated TLR2/MyD88/IL-8 and exacerbated inflammation in mycobacteria (*Mycobacterium avium paratuberculosis*)-infected macrophages, providing important insights to understand the effect of nicotine on worsening inflammation symptoms in patients with Crohn’s disease (50). Caution should be exercised in nicotine administration due to organ specificity.

To sum up, based on pathological parameters, inflammatory markers, and histological features, oral administration of nicotine is a very efficacious way for reversing colitis in rodent models. Concerning Crohn’s disease, there are several contradictory results, especially in the TNBS rat model when the concentration is >2.5 mg/kg. Al-Sharari (31) compared the different routes of nicotine administration in DSS-induced colitis models; low, but not high, doses of oral nicotine had a better effect than chronic s.c. or minipump infusion of the drug. Oral nicotine at 25 µg/ml for 10 days and the corresponding 18-ng/ml plasma concentrations had the best protective roles in male C57BL/6 mice. These findings indicate that nicotine seems to target and affect the colon locally when administered orally, with a marginal therapeutic index. However, the way nicotine is added to drinking water makes quantification difficult. In total, 0.1–1 mg/kg/day (p.o.) or 0.1–8 mg/kg/day (s.c.) or 0.25–6 mg/kg/day (i.p.) for mouse might be the effective range of nicotine in inflammatory bowel disease. In a word, nicotine’s anti-inflammatory effects seem to explain the beneficial outcome of tobacco smoking on ulcerative colitis activity.

### 3.2 Arthritis

Based on the definition by the Centers for Disease Control and Prevention, National Center for Chronic Disease Prevention and Health in the USA (2019), arthritis is any disorder that affects the joints, including joint pain and stiffness, usually accompanied by redness, warmth, swelling, and decreased range of motion of the affected joints, which typically worsens with age. There are >100 types of arthritis. The most common types of arthritis are rheumatoid arthritis and osteoarthritis (51). Nicotine tends to exert positive effects on rheumatoid arthritis and osteoarthritis.

#### 3.2.1 Anti-Inflammatory Effect of Nicotine on Arthritis

Rheumatoid arthritis is an immune-mediated inflammatory disease of unknown etiology, characterized by inflammation of the synovium, leading to the destruction of cartilage and bone. Rheumatoid arthritis has affected approximately 24.5 million people globally as of 2015. Its symptoms usually last over weeks and months (52). In animal experiments, collagen induction can increase arthritis scores, lesions on the cartilage, cellular infiltration, or bone destruction, typically in DBA/1 mice, sharing common characteristics with rheumatoid arthritis in humans. In male DBA/1 mice, oral administration of nicotine (50 μg/ml in drinking water) 4 days before induction ameliorated collagen-induced arthritis (CIA), which was exacerbated by vagotomy (day 20 to 47) (53). In the CIA male DBA1/J mice model, oral nicotine (2 mg/kg, day 21 to 28) reduced the clinical arthritis scores, decreased the number of infiltrated cells, and rescued the lesions on the cartilage. Until day 35, the nicotine group significantly suppressed histological IL-17A, serum TNF-α, and pro-inflammatory cytokines IL-17A and IL-6 mRNA expression in isolated splenocytes. Furthermore, isolated CD4^+^IL-17A^+^Th17 cells expressing α7 nAChR stimulated by nicotine may decrease their pro-inflammatory function (16). Nicotine was added to drinking water (100 μg/ml, about 300 μg nicotine per day per mouse) for male DBA/1 mice from the day of immunization by chicken collagen II, resulting in a delay in arthritis. In addition, nicotine (10 μg/ml) was further shown to suppress the production of IL-6, not TNF-α, by splenocytes stimulated by LPS (54). Moreover, an intraperitoneal injection of nicotine also effectively and safely alleviated arthritis. In a male DBA/1 mice CIA model, the concentration of nicotine (400 μg/kg, i.p.) for 7 days also reduced synovial inflammation by inhibiting bone degradation and reducing TNF-α expression in synovial tissue and plasma (53). Intraperitoneal pretreatment with nicotine daily (250 µg/kg) decreased synovial proliferation, inflammatory monocyte/macrophage infiltration, and bone destruction, depending on the downregulation of macrophage inflammatory protein−1α, MCP−1, and C−C chemokine receptor (CCR)2 (55). The nicotine group (250 µg/kg, i.p.) significantly attenuated clinical arthritis for 33 days after the first immunization. Nicotine alleviated CIA inflammation by inhibiting Th17 cell response, which is the main target in rheumatoid arthritis, mainly embodied in a decrease in Th17-related transcription factor (RORγτ) expression in the spleen and IL-17A activities in serum. In addition, Th2 cells, which were considered an anti-inflammatory Th subtype, were evoked by nicotine with reduced IL-4 levels in serum and increased GATA3 levels in the spleen to weaken CIA for reducing joint swelling and pain, reducing infiltration of inflammatory cells, alleviating synovial inflammation, and reducing bone destruction simultaneously (56). Furthermore, nicotine decreased not only the early pro-inflammatory factors TNF-α and IL-6 levels in serum but also the late powerful pro-inflammatory factors high-mobility group box chromosomal protein 1 (HMGB1) expression and nuclear translocation in the inflamed joints. However, nicotine did not affect the anti-inflammatory factor IL-10 expression in serum, similar to ulcerative colitis as mentioned before (57). In fibroblast-like synoviocytes isolated from rheumatoid arthritis patients undergoing joint replacement surgery, 0.1–100 µM of nicotine did not affect cell viability, but the 1–10-mM range induced significant cytotoxicity. Nicotine (0.1, 1, and 10 µM) reduced the expression of IL-6 and IL-8 and the activity of their upstream NF-κB induced by TNF-α (20 ng/ml) (58). Furthermore, it was reported that the intracellular JAK2-STAT3-IL-6/MCP-1 signaling mechanism of the anti-inflammatory pathway of nicotine (10 μM) in TNF-α-stimulated fibroblast-like synoviocytes isolated from patients is a major constituent of the hyperplastic synovium, with an important role in joint inflammation and bone destruction observed in rheumatoid arthritis (59). A non-STAT-dependent pathway was discovered in nicotine in CIA synovial tissues in DBA/1 mice and patients, derived from fibroblast-like synoviocytes and mediated by the suppressor of cytokine signaling pathway SOCS3. Nicotine pretreatment (250 μg/kg/day, i.p.) attenuated clinical arthritis and histopathology findings by decreasing the SOCS3 protein levels and its downstream factors IL-6 (60). In both TNF-α-induced fibroblast-like synoviocytes and LPS or IFN-γ-treated monocytic cell lines (U937), nicotine (0.1, 1, and 10 μM) inhibited cell proliferation and reduced the expression of MMP9 and VEGF, indicating that nicotine played a crucial protective role in pannus formation and joint destruction in rheumatoid arthritis (61). In addition to DBA/1 mice, rheumatoid arthritis was induced in male Wistar rats by intradermal injection into the hind paw of 0.1 ml of complete Freund’s adjuvant, containing 10 mg/ml of killed *Mycobacterium*. In these rats, nicotine (2.5 mg/kg, orally, for 11 days) suppressed the arthritis index of hind paw swelling and weight gaining and decreased some hematological and biochemical parameters, including rheumatoid factor, C-reactive protein, NO, MPO, IL-1, and IL-17. Furthermore, the combination of 1.25 mg/kg nicotine and 50 mg/kg thymol (orally) resulted in a synergistic benefit and tolerable safety margin in alleviating this rheumatoid arthritis model (62). In rheumatoid arthritis patients, although whole blood from rheumatoid arthritis patients with high C-reactive protein and HMGB1 levels showed reduced vagus nerve activity when stimulated by LPS, nicotine (100 µM) attenuated the release of inducible cytokine TNF, implicated in the pathogenesis of rheumatoid arthritis (63).

Osteoarthritis is a degenerative joint disease that causes chronic disability among the elderly. Despite recent advances in the symptomatic management of osteoarthritis by pharmacological and surgical approaches, there is a lack of optimal approaches to manage inflammation in the joints, which causes cartilage degradation and pain. Monosodium iodoacetate induction osteoarthritis mice model is a standardized osteoarthritis model. In the C57BL/6J mouse model of osteoarthritis induced by an injection of monosodium iodoacetate into the knee joint, nicotine (0.5 or 1 mg/kg once daily, i.p., 1 week) reduced mechanical allodynia, cartilage degradation, and the upregulation of MMP9 production by macrophages *via* α7 nAChR through modulation of the PI3K/Akt/NF-κB pathway (64). In monosodium iodoacetate-induced osteoarthritis of male Sprague–Dawley rats, the systemic administration of nicotine (1 mg/kg once per day for 5 weeks, i.p.) alleviated joint degradation. Accordingly, in isolated primary cultured rat chondrocytes, pretreatment with nicotine (10 µM) suppressed MAPK (p38/Erk/JNK) phosphorylation and phosphorylated NF-κB activation induced by IL-1β or monosodium iodoacetate *via* α7 nAChRs (65). With the small-scale study of 16 male Lewis rats using another standardized osteoarthritis model with monoiodoacetate, nicotine (0.625 mg/kg, i.p., 42 days) did not result in significant differences but still showed promising tendencies in cartilage degeneration (~36% restoration), also indicating that nicotine may be an additional treatment approach for osteoarthritis (66). In surgically created osteoarthritis mice, nicotine also exhibited a potential therapeutic effect. In primary chondrocytes of meniscectomy osteoarthritis of C57BL/6 mice model, nicotine (1, 10, and 100 µM) decreased the IL-1β-induced IL-6 and MMP3/MMP13 expression in a dose-dependent manner in the wild type but not in α7 nAChR^-/-^ type, indicating that nicotine’s effect is also mediated by α7 nAchR in this non-neuronal cholinergic system (67). In a male Sprague–Dawley rat model of early-stage osteoarthritis induced by immobilizing the left knee joints, nicotine (50 μg/ml) added to the drinking water ameliorated cartilage damage, promoted matrix production, and had an anti-inflammatory effect by reducing the serum levels of TNF-α and the expression of TNF-α in the synovial tissue *via* stimulating α7 nAChR, indicating that nicotine may have potential as a therapeutic strategy for early osteoarthritis (68).

#### 3.2.2 Pro-Inflammatory Effect of Nicotine on Arthritis

The first study on the aggravation of arthritis by nicotine was reported by Yu et al. in 2011 (69). They found that, in heat-killed *M. tuberculosis* H37Ra (Mtb)-induced arthritis of Lewis rat models, pretreatment with nicotine (0.625, 1.25, or 2.5 mg/kg, i.p.) before induction of arthritis exacerbated the disease. In contrast, post-injection of nicotine after the onset of arthritis decreased the disease severity. Thus, the critical variables in the opposite outcomes, including the timing of nicotine administration and the induction model, differed quantitatively and/or qualitatively from that of male rats. In female ovariectomized BALB/C mice immunized with chicken collagen II in complete Freud’s adjuvant injected in the tail root, nicotine (0.03%) in drinking water caused arthritis by supporting the non-exhausted PD-1^-^IL-7R^+^CD8^+^ T cells, possibly by inducing the release of survivin protein from the bone marrow (70). The decreased secretion by nicotine (10^−8^ to 10^−7^ M) in a high concentration of MMP1/MMP13 and two proposed markers of osteoarthritis, fibronectin (FINC) and chitinase 3-like protein 1 (CHI3L1), in primary chondrocytes from patients also reminded us of the cautious and judicious use of nicotine in osteoarthritis treatment (71).

To sum up, these diverse dose-dependent effects of nicotine demonstrate the need for specific and strict dose–response studies on disease models to clarify all the controversial data on the mechanism of this drug. Altogether we still consider that even if nicotine could have some beneficial effects on cartilage, the multiple risks related to smoking far outweigh the conditional beneficial effects of nicotine. However, nicotine is just one of the compounds found in tobacco smoke; therefore, the role of the other ones cannot be ruled out. In treating arthritis, we suggest that injecting nicotine in the lesion site may be a better direction in future studies.

### 3.3 Oral Diseases

Tobacco smoking has been established as a significant risk factor for periodontitis, oral mucosal changes, gingival recessions, and root surface caries (72). This is different from treating colitis and arthritis; nicotine also worsens inflammation in the oral cavity, similar to smoking. However, with the deepening of research, nicotine interestingly relieves oral inflammation under certain conditions.

#### 3.3.1 Anti-Inflammatory Effect of Nicotine on Oral Diseases

A new *in vitro* study on human oral epithelial cell HSC-2 line showed that nicotine (10^-8^–10^-3^ M), for a short time (24 h), could exert a suppressive effect on the production of inflammatory mediators, including IL-8 and ICAM-1 induced by LPS or TNF-α, indicating that nicotine may serve as an anti-inflammatory agent. However, nicotine also inhibited β-defensin induced by *Porphyromonas gingivalis* LPS, indicating that it may allow the overgrowth and invasion of potential periodontal pathogens. Importantly, nicotine did not affect HSC-2 proliferation/viability in this concentration range. It is impossible to ignore that nicotine may have a strong toxic effect on different cell types at concentrations higher than 10^−3^ M (73). It seems that the oral microbiota may regulate the effect of nicotine.

#### 3.3.2 Pro-Inflammatory Effect of Nicotine on Oral Diseases

Many studies have shown the pro-inflammatory effect of nicotine on oral diseases. This part mainly reviewed periodontitis, gingivitis, pulpal inflammation, oral mucosal inflammation, denture inflammation, *etc.*

Periodontitis is the leading cause of tooth loss in adults, resulting from the interaction of the host’s defense mechanisms with bacteria accumulating on the tooth surface (74). Inflammatory responses and osteoclastogenesis are commonly involved in the development of periodontitis. Two *in vivo* studies are available on the aggravation of periodontitis by nicotine. In an *in vivo* study, 5-week-old male Wistar rats intraperitoneally injected with nicotine (0.7 mg/kg for 30 days) exhibited decreased levels of bone alkaline phosphatase and osteocalcin as important markers for bone mineralization and activity of osteoblasts, with increased TNF-α and COX−2 expression levels causing ischemia and inflammation of the periodontium and higher alveolar bone loss. Here catalpol was shown to improve nicotine-induced injury to the alveolar bone by promoting alveolar bone mineralization and inhibiting the inflammatory factors (75). Another *in vivo* study on periodontitis-susceptible Fischer 344 rats injected with nicotine in the neck skin (0.8 mg/kg for 3 weeks) and intraperitoneally injected with LPS (100 μg/kg) found that these developed significantly more periodontal bone loss and periodontal tissue destruction, while nAChR antagonist mecamylamine abolished this effect. Nicotine treatment alone indirectly reduced TNF-α, TGFβ, and IL-10 production, indicating that the acetylcholine receptors activated by nicotine may suppress protective immune responses through the cholinergic anti-inflammatory pathway by altering the immune system responses and the susceptibility to periodontitis (76). In addition, this part of the research mostly focused on human periodontal ligament cells induced by nicotine (5 mM) and/or LPS (1 µg/ml) *in vitro*. Under this condition, pretreatment of overexpressed zinc finger protein A20 using a recombinant adenovirus encoding A20 inhibited the inflammatory and osteoclastogenic effects of nicotine and/or LPS by inhibiting the PKCa, Akt, GSK-3β, ERK, and NF-κB pathways, including downstream cytokines COX-2, TNF-a, IL-1β, IL-6, and IL-17 and especially RANKL, a receptor activator of nuclear factor kappa-β ligand, which mediates osteoclast differentiation (77). LPS plus nicotine stimulated the levels of pro-inflammatory medium PGE2, NO, TNF-α, IL-1β, IL-6, IL-12, and MMPs (MMP1, MMP2, and MMP9) and suppressed multiple ECM molecules (collagen I, elastin, and fibronectin) by PI3K/GSK3β/MAPK (P38, JNK, ERK)/NF-κB and β-catenin signaling pathways. Here resistin showed the regulation of these factors and exerted anti-inflammatory effects on this chronic periodontal disease cell model (78). Nicotine plus LPS likewise upregulated iNOS, COX-2, NO, PGE2, TNF-α, IL-1β, IL-8, some TRAP (+) cells, and resorption *via* PI3K/PKC/Akt/MAPK (P38, JNK, ERK) and the NF-κB/c-Fos/NFATc1 signaling axis, involved in inflammatory bone destruction and osteoclast differentiation, with an increase in phospholipase D (PLD) 1 and PLD2 isoforms (79). When human periodontal ligament cells were cocultured with CD4^+^ T cells, nicotine (10^-5^ M, 24 h) dramatically repressed cell viability and increased apoptosis, which was associated with the collapse of periodontal tissues and elevated matrix metalloproteinases MMP1, MMP3, and cytokines IL-1β, IL-6, IL-17, IL-21, and chemokines CXCL12. The receptor α7 nAChR played a major mediating role, and its inhibitor α-bungarotoxin reversed nicotine-induced apoptosis, increased viability, and attenuated the nicotine-triggered production of these factors (80). In addition to inflammatory cytokines and osteoclast markers, other molecules may also participate in nicotine-induced periodontitis. In human periodontal ligament cells, hypoxia-inducible factor-2α (HIF-2α) was upregulated in a dose- and time-dependent manner. HIF-2α inhibitors attenuated the 10-mM-nicotine- and 1-µg/ml-LPS-induced production of NO and PGE2 and upregulation of iNOS, COX-2, pro-inflammatory cytokines (IL-1β, TNF-α, IL-1β, IL-6, IL-8, IL-10, IL-11, and IL-17), and matrix metalloproteinases (MMP1, 8, 13, 2 and 9) and reversed the effect on TIMPs (TIMP1 and 2) *via* Akt, JAK2 and STAT3, ERK and JNK MAPK, NFκB, c-Jun, and c-Fos. Thus, HIF-2α was positively associated with inflammatory responses and osteoclast differentiation induced by nicotine (81). Another protein, peptidyl-prolyl cis/trans isomerase NIMA-interacting 1 (PIN1), was upregulated in chronically inflamed human periodontal ligament cells in periodontitis patients. The inhibition or knockdown of PIN1 markedly attenuated 1-µg/ml-LPS- and 5-mM-nicotine-stimulated NFκB/COX-2 and iNOS/PGE2, and the NO pathway also attenuated the increased number of tartrate-resistant acid-phosphatase-stained osteoclasts, osteoclast-specific gene RANK expression, and osteoclastogenic cytokine expression (TNF-α, IL-1β, IL-6, IL-11, IL-17, and IL-23, not IL15) (82). However, 1-mM nicotine treatment alone did not upregulate PIN1, while 5 and 10 mM nicotine could significantly increase the PIN1 protein level (82). HO-1 was also confirmed to be involved in 5-mM-nicotine- and 1-µg/ml-LPS-induced inflammatory effects on human periodontal ligament cells by Pi et al. (83). Treatment with an HO-1 inhibitor and HO-1 siRNAs blocked the stimulated NO and PGE2 release as well as the expression of iNOS and COX-2 (83). Furthermore, during the treatment of apigenin on this cell models, LPS (1 µg/ml) and nicotine (5 mM) induced HO-1 protein expression and activities, further upregulating iNOS, COX-2, NO, PGE2, IL-1β, TNF-α, IL-6, and IL-12 by the PI3K/MAPK (P38, JNK, ERK)/PKC signaling pathway, indicating that apigenin possesses anti-inflammatory activity for the prevention and treatment of periodontal diseases associated with smoking and dental plaque (84). Also, *via* the HO-1 pathway, carbon monoxide (CO)-releasing molecule-3 pretreatment suppressed the inflammatory and osteoclastogenic cytokines, including PGE2, COX-2, and RANKL/OPG ratio by releasing CO (85). Zhou et al. (86) found that the activation of GSK-3β played indispensable roles in nicotine-induced periodontitis using human periodontal ligament stem cells (PDLSCs). Nicotine (10^−9^ M) alone did not affect the osteoblastogenesis/osteoclastogenesis balance of PDLSCs. However, it significantly exacerbated the destructive effect of inflammatory factors (5 ng/ml IL-1β and/or 10 ng/ml TNF-α) by decreasing the osteogenic differentiation indicators ALP, RUNX2, BSP, and OCN, increasing the osteoclast-forming indicators RANKL/OPG ratio by suppressing the expression of phosphorylated GSK-3β, and diminishing the function of its downstream factor α7 nAChR. The apoptotic effect exerted by nicotine on these first-line defensive cells, such as polymorphonuclear cells (PMNs) and mononuclear cells, may be an important feature of the pathogenesis of the periodontal disease. A brief exposure of PMN to nicotine concentrations ranging from 0.01 to 0.3% shortened the accelerated cell apoptosis in a dose-dependent manner. Concerning mononuclear leukocytes, nicotine could not induce apoptotic modifications on cells observed up to 72 h of culture time. However, it inhibited IL-1β release and procoagulant activity expression, indicating that when mononuclear cells become the predominant population in inflamed tissues, nicotine could offer an interesting exogenous example of the phlogistic balance that governs the evolution and resolution of the persistent inflammation in periodontal disease (87). Thus, the double-edged sword feature of nicotine necessitates more detailed research.

Specifically, in gingivitis, 1 mM, not 0.1 mM, nicotine stimulated the production of IL-1α in gingival keratinocytes from healthy, non-tobacco-using subjects. When combined with *Escherichia coli* and *P. gingivalis* LPS (10 µg/ml), the release of IL-1α and IL-8 cytokines increased significantly. These findings support an interactive effect of smoking and bacterial factors on host response in periodontal disease (88). The exposure of human gingival fibroblasts to nicotine (2.5, 10, and 15 mM for 2–24 h) resulted in a significant increase in COX-2 mRNAs and protein expression (89). Furthermore, in this cell line, nicotine (5 mM) and LPS (1 µg/ml) upregulated the mRNA and protein expression of sirtuin 1 (SIRT1) as well as ROS, PGE2, TNF-α, IL-1β, IL-6, IL-8, and IL-17 through the PKC, PI3K, MAPK (P38, JNK, ERK), and NF-κB pathways. SIRT1 activation and over-expression by resveratrol and adenovirus encoding SIRT1 have potent cytoprotective and anti-inflammatory effects on nicotine- and LPS-induced cytotoxicity, oxidative stress, and pro-inflammatory activities (90). Nicotine (0.3 and 1 mM) significantly reduced human β-defensin-2 mRNA and protein expression and significantly increased IL-8 production, compared with cultures with no nicotine treatment, in response to *P. gingivalis* LPS and TNF-α-induced human gingival epithelial cells from subjects with clinically healthy periodontium and no history of periodontitis (91).

Concerning oral mucosal inflammation, nicotine was also used as an inducer to generate neurogenic inflammatory reactions. In the human oral mucosal keratinocyte 100 cell line, nicotine (1 µM and 1 mM) did not influence cell viability but stimulated the concentrations of periodontal inflammation and oral pain correlation factor substance P (SP), a major mediator of neurogenic inflammation, and IL-1β. Here neutral endopeptidase (NEP) exerted a good relieving effect by downregulating the SP and IL-1β levels by cleavage (92). Nicotine (EC_50_ 557 μM) potentiated capsaicin-evoked immunoreactive calcitonin gene-related peptide release in a concentration-dependent manner by α3, α4, or α6 nAChR subunit in trigeminal ganglia which co-localized with CGRP-/VR1 (the capsaicin receptor) in adult male Sprague–Dawley rats. These findings suggest that nicotine may predispose the buccal mucosa to an enhanced neurogenic inflammatory response to different stimulatory factors, such as thermal, chemical, and mechanical tissue damage or infection (93). One *in vitro* experiment also showed that 10 mM nicotine did not affect the TNF-α-stimulated tissue and un-inflamed reconstituted human epithelium model and did not significantly affect the release of IL-1α, IL-6, IL-8, and GM-CSF cytokines after 5 min and 24 h, respectively (94). This suggests that nicotine may promote the existing inflammation, not healthy oral mucosa cells, for a short time. In addition, systemic nicotine treatment (nicotine sulfate, 2 mg/kg, subcutaneously, daily for 28 days) induced inflammatory leukocyte infiltration and cellular desquamation, blood vessel dilation, hemorrhage, and epithelial degeneration in the rat tongue mucosa (95).

Denture stomatitis is a condition of painless inflammation and erythema of denture-bearing mucosa. Nicotine increased the growth of *Streptococcus mutans* and *Candida albicans* in denture biofilms. Furthermore, 4 mg/ml nicotine in tryptic soy broth significantly increased the coaggregation of these two microorganisms after 24 h of incubation, increasing the likelihood of denture stomatitis and caries (96).

The matrix metalloproteinase enzymes are key participants in pulpal inflammation. When the mineralized matrix of a tooth is damaged, the pulp is exposed to a plethora of environmental stimuli. In human dental pulp cells, Manuela et al. (97) showed that nicotine (0–50 μM) stimulated MMP2 and MMP28 gene expression extracted from impacted third molars of healthy patients. In addition, nicotine (5, 10, and 50 μM) can increase human dental pulp cell proliferation through nicotinic cholinergic receptors and downstream MAPK signaling pathways.

To sum up, the 5-mM concentration of nicotine has mostly been used in many previous studies. The range of nicotine concentrations measured in the saliva of long-term snuff users is 0.6–9.6 mM (98). From the experimental data, when the nicotine concentration is >1 mM, it is more likely to cause oral cavity inflammation; when the concentration is lowered down to 0.01 μM, it may exert a beneficial inhibitory effect on oral cells. To date, overwhelming data indicate that nicotine’s dual regulation of the immune system through cytokines and nAChR accentuates oral inflammation, especially in the presence of oral pathogenic bacteria. The data are insufficient, especially *in vivo*. However, the recent research also indicates that the medicinal value of nicotine when treating exposed oral cells at low concentrations (10^-8^–10^-3^ M) should be explored and clarified.

### 3.4 Skin Diseases

#### 3.4.1 Anti-Inflammatory Effects of Nicotine on Skin Diseases

The evidence on smoking and skin disease is conflicting. However, the potential for nicotine treatment is in skin conditions that occur less often in smokers than in non-smokers.

An interventional study early in 1997 showed that nicotine patches suppressed the cutaneous inflammatory response to sodium lauryl sulfate and ultraviolet radiation and transiently suppressed reactive hyperemia following arterial occlusion in 10 lifelong non-smokers (99). Ultraviolet radiation exposure is a common and well-characterized method of inducing inflammation in the skin. At the end of the 6-week oral nicotine treatment in drinking water (from 25 to 50 μg/ml on day 3, 100 μg/ml on day 5, and the final concentration of 150 μg/ml on day 7, maintained for 6 weeks), female C57BL/6 mice were exposed to ultraviolet radiation to induce inflamed redness in a thickening skin model. Both RNA and protein production of IL-1β, not IL-6 and TNF-α, decreased significantly in the skin treated with nicotine by regulating α7 nAChR and SOCS3. It was demonstrated that the oral administration of nicotine altered inflammation in the skin, independent of vagal innervation (100).

In Bechet’s disease, nicotine maintained a specific anti-inflammatory effect on keratinocytes (isolated from normal neonatal foreskin specimens, which were removed during circumcision) and endothelial cells (HMEC-1) in the serum of patients. After keratinocytes and HMEC-1 were incubated with the serum of 20 patients with Behçet’s disease and 20 healthy controls for 4 h, nicotine (6 μM) significantly decreased IL-8 and IL-6 released by HMEC-1 maintained in both patients’ and controls’ sera. However, only IL-6 released by keratinocytes was maintained in patients’ sera. VEGF released by both cells increased markedly after nicotine treatment in either serum. Thus, it is likely that nicotine could suppress neutrophil-mediated inflammatory actions (101).

In the passive cutaneous Arthus reaction, an acute inflammatory response resulting from the deposition of immune complexes in the tissue, the plasma extravasation, and polymorphonuclear leukocyte infiltration serve as indexes to evaluate the anti-inflammatory activities. Kubo et al. (102, 103) reported that nicotine (0.4 mg/kg injected subcutaneously or 0.8-mg/kg intraperitoneal injection) inhibited the plasma exudation in the passive cutaneous Arthus reaction in Wistar male rats by suppressing NO production in polymorphonuclear leukocyte primed with TNF-α by elevating the blood corticosterone levels. It is well established that nicotine stimulation of the brain’s nicotinic acetylcholine receptors is related to the nicotine-induced inhibition of the passive cutaneous Arthus reaction.

#### 3.4.2 Pro-Inflammatory Effect of Nicotine on Skin Diseases

Nicotine [10^-8^ M and 10^-4^ M, three injections of 20 μl (total = 60 μl) around the wounds once daily for 3 days] injected into full-thickness excisional skin wounds of female C57BL/6 mice minimally affected the inflammatory mediators like TNF, IL-6, and IL-12. However, it downregulated the expression of growth factors like VEGF, PDGF, TGF-β1, and TGF-β2 and the anti-inflammatory cytokine IL-10, reflecting the overall detrimental effects of tobacco on wound healing and general skin diseases (104).

### 3.5 Multiple Sclerosis

#### 3.5.1 Anti-Inflammatory Effect of Nicotine on Multiple Sclerosis

Multiple sclerosis (MS) is a demyelinating autoimmune disease characterized by inflammation, demyelination, and neurodegeneration within the central nervous system. The cause of MS is still unknown, and there is no cure for it. Nicotine exhibited protective effects in an experimental model of MS and might be recommended as a promising drug (105). In two Swedish population-based, case–control studies (7,883 cases; 9,437 controls), subjects with different snuff use habits had a decreased risk of developing MS, suggesting that nicotine might exert anti-inflammatory and immune-modulating effects (106). In relapsing–remitting MS patients, compared to healthy donors, nicotine (10 μM, 24 h) and treated peripheral blood mononuclear cells stimulated by phytohemagglutinin (20 μg/ml) had a significant decrease in mRNA and protein levels of IL-1β and IL-17 *via* α7 nAChR mediation to down-modulate the inflammation in MS (107).

Experimental autoimmune encephalomyelitis is the most commonly used animal model for MS. Peripheral monocyte/macrophage recruitment, microglial activation, iNOS upregulation, and pro-inflammatory cytokine production are important mechanisms for inducing experimental autoimmune encephalomyelitis and determining disease severity. Furthermore, 200 μg/ml nicotine hydrogen tartrate salt in drinking water induced mild, delayed, and chronic clinical signs and less inflammation in MOG_35-55_-induced female C57BL/6 mice (108). Nicotine (28 days, infusion rate of 0.25 μl/h, mini-osmotic pumps) was protective against experimental autoimmune encephalomyelitis by reducing nestin expression, partially allowing for the proliferation (Ki67^+^) of ependymal cells in areas of inflammation and increasing mature anti-inflammatory M2 subtype microglia (NG2^+^ and CC1^+^) to promote disease recovery (105, 109). Nicotine also distinctively affected microglial viability, activation, and function, contrary to non-nicotine cigarette components, especially acrolein that has cytotoxic effects on microglia (105). In female Wistar rats with experimental autoimmune encephalomyelitis induced by guinea pig spinal cord homogenate, both nicotine (2.5 mg/kg, i.p.) and mesenchymal stem cells (2 × 10^6^ cells per rat) exhibited a more desirable outcome, causing the regression of the average mean clinical score and neuropathological features, by increasing the IL-10 levels, thus decreasing the IL-17, TNF-α, and IFN-γ levels of splenocytes; the combination therapy was more favorable than the treatment with either therapy alone (110). α7 nAChRs, but not α9 nAChRs and β2 nAChRs, are involved in nicotine-dependent protection against experimental autoimmune encephalomyelitis. However, it is noteworthy that nAChR α9 and β2 play different controversial roles in modulating immune functions in experimental autoimmune encephalomyelitis (111, 112). At present, there is no conclusive evidence about the worsened function of nicotine in multiple sclerosis.

### 3.6 Sepsis and Endotoxemia

#### 3.6.1 Anti-Inflammatory Effect of Nicotine on Sepsis and Endotoxemia

Sepsis is a complex clinical syndrome resulting from a severe inflammatory response to infection. Therefore, the control of inflammation is crucial to minimize sepsis-related deleterious effects. Several mediators and cell types participate in the pathophysiology of sepsis; however, the available therapeutic strategies are far from satisfactory. Activation of nicotinic α7 nAChRs by nicotine leads to improved survival rate and multiple organ dysfunction syndromes in an experimental model of sepsis under different induction conditions as detailed in the following discussion.

In experimental abdominal sepsis induced by an intraperitoneal injection of live *E. coli*, the female C57BL/6 mice pretreated with nicotine (100 µg/ml in drinking water) or a combination of vagotomy displayed lower levels of TNF-α, IL-6, and IL-1β in peritoneal lavage fluid and plasma. It also exhibited decreased liver injury with lower AST and ALT in the vagotomy mice, confirming that nicotine acts on the peripheral part of the cholinergic anti-inflammatory pathway, independent of the integrity of the vagus nerve (113).

In the LPS-induced male Wistar rat endotoxemia model by tail vein injection, nicotine (0.1 mg/kg) treatment, in the same way, improved the survival rate by 40%, lowered the elevation of ALT and creatine kinase-MB (CK-MB) and the serum levels of TNF-α and IL-6 significantly, recovered the plasma diamine oxidase (DAO) activity, and had little influence on IL-10 levels (114).

The cecal ligation and puncture (CLP)-induced female C57BL/6 mouse model is a clinically relevant animal model for human sepsis because it causes lethal peritonitis produced by polymicrobial infection. In this model, nicotine (400 µg/kg, i.p.) significantly reduced the lethality for 3 days (115). In male BALB/C mice receiving a dose of LPS by intraperitoneal injection or/and CLP, nicotine (400 µg/kg, i.p.) also prevented lethal endotoxemia and clinical manifestations, including lethargy, diarrhea, piloerection, huddling, hypothermia, and hematocrit decrease. It had no protective effects when the nicotine concentration was low, *i*.*e*., up to 40 µg/kg (116). Nicotine inhibited the NFκB activity and upregulated the HO-1 levels in the heart and lungs and then mediated HMGB1 translocation to the cytoplasm; subsequently, extracellular release in blood played major protective roles *in vivo*. Furthermore, the verification mechanism in macrophage RAW264.7 induced by the LPS inflammatory cell model showed that nicotine activated α7 nAChR and subsequently increased the Ca^2+^ influx. The increased Ca^2+^ levels led to the activation of classical PKCs, which, in turn, stimulated ROS production *via* NADPH oxidase. The increased ROS levels activated the PI3K/Akt/Nrf2 pathway, culminating in HO-1 induction in macrophages, which resulted in anti-inflammatory action, indicating decreased pro-inflammatory TNFα, iNOS, and HMGB1 activities (116, 117). Furthermore, Kim et al. reported that intraperitoneal nicotine (50, 100, 200, and 400 μg/kg), after induction by LPS and CLP, improved the sepsis-induced mortality, attenuated liver and lung failure, and suppressed the inflammatory cytokines TNF-α, IL-1β, and IL-6. This protective effect of nicotine can be associated with nicotine-suppressed Toll-like receptor 4 protein expression and PU.1 activity through the α7 nAChR/PI3K signaling pathway (118). In human macrophages, nicotine (100, 1, and 10 μM) also upregulated the expression of IL-1 receptor-associated kinase M (IRAK-M), a negative regulator of innate TLR-mediated immune responses, a single (JAK2/PI3K/STAT3) or two convergent cascades (JAK2/STAT3 and PI3K/STAT3), to inhibit LPS-induced TNF-α production. However, a persistent IRAK-M overexpression may lead to the immune cells’ tolerance to nicotine (119). The protective effects of nicotine in lethal sepsis required the spleen, showing that decreased HMGB1 in the serum of CLP male BALB/C mice by nicotine (400 μg/kg, i.p.) was eliminated by splenectomy (120). TNF production through spleen macrophages in the red pulp and the marginal zone was specifically attenuated with the electrical stimulation of the vagus nerve in male Sprague–Dawley rats and nicotine (2 mg/kg, i.p.) treatment in the LPS-induced endotoxemia model of male BALB/C mice during endotoxemia (15). Thus, the cholinergic anti-inflammatory pathway between the vagus nerve and the splenic nerve can allow the rapid and precise control of systemic cytokine production and the trafficking of inflammatory cells to distant sites (15, 120). In more detail, microsomal prostaglandin E synthase-1 gene and PGE2 in the spleen have a crucial role in bridging the immune and nervous systems influenced by nicotine *in vitro* (121).

Sepsis was induced in outbred albino mice of both genders by an intraperitoneal injection of the 24-h culture of *E. coli* (2.5 × 10^9^ microbial bodies). Nicotine (17.5 ± 2 mg/kg, 7 ± 0.8 mg/kg, s.c.) pretreatment reduced the mortality during the early phase of sepsis and reduced the concentrations of TNF-α, IL-1β, IL-6, and MIP-2 after 2 or 10 h of sepsis. These animal survival rates in sepsis may be due to the stimulation of the phagocytic monocytic system of mAChR cells in the liver, gastrointestinal tract, and spleen, leading to increased phagocytic and metabolic activity (122, 123). Male Wistar albino rats of CLP sepsis received chronic tap water with (5 mg/kg) nicotine for 14 days and an acute injection of nicotine (30 mg/kg, i.p.) for 5 days, resulting in the recovery of histologically observed injury, lipid peroxidation, and myeloperoxidase activity and the prevention of GSH depletion, indicating that nicotine was advantageous in regulating systemic inflammation by balancing the oxidant and antioxidant systems (MPO, GSH, and MDA). In addition, chronic treatment seems to be better than the acute treatment by nicotine after evaluating the oxidative injury and morphological organization of lung and liver parenchyma, cardiac muscle, ileal mucosa, glomeruli, and tubules, significantly the lung tissue (124), although another inconsistent study showed that nicotine (400 µg/kg, i.p.) had caused more lung damage by increasing the neutrophil polymorphonuclear infiltration in CLP-treated peritonitis of male Wistar rats (125). This may have to do with the severity of the disease and the tolerance of the animal species. However, nicotine appeared to be protective to heart abnormalities because LPS-evoked hypotensive and tachycardic responses, reductions in time-domain indices of heart rate variability and spectral measure of cardiac sympathovagal balance, and upregulation of serum TNF-α were reversed by nicotine (25, 50, and 100 μg/kg, i.v.) *via* α7 and α4β2 nAChRs in male Wistar rats with endotoxemia (126). Furthermore, in isolated left kidney with the addition of acetylcholine and 5′-N-ethylcarboxamidoadenosine from a Wistar rat endotoxemia model induced by LPS, nicotine (2 mg/kg, i.p.) offset the LPS facilitation of renal vasodilation and inflammation through the HSP70/TNFα/iNOS signaling pathway *via* α7- and α4β2-type nAChRs (127).

The basal levels of acetylcholine are important to modulate sepsis-associated inflammatory responses. In the CLP sepsis model and vesicle acetylcholine transporter knockdown (VAChT^KD^, C57BL/6J background), mutant mice with less vesicular Ach transporter protein contributed to significantly increased TNFα levels, higher levels of bacteria, and reduced neutrophil accumulation and CXCL2 in the peritoneal cavity. This phenotype was reversed by nicotine salt (400 μg/kg, i.p.) treatment (128).

To sum up, despite the health risks of toxic and/or carcinogenic chemicals in tobacco, nicotine appears to have beneficial effects on systemic inflammatory responses. In the deadly disease of sepsis and endotoxemia, nicotine may be helpful for an emergency patient who requires life-saving care, although some side effects were inescapable for organs. Clinical studies that include nicotine supplementation are required to further elucidate the therapeutic potential of nicotine in sepsis and endotoxemia.

#### 2.6.2 Pro-Inflammatory Effect of Nicotine on Sepsis and Endotoxemia

One study showed the adverse effects of nicotine (400 µg/kg, i.p.) when given after treatment in a male Wistar rat CLP model of sepsis (125). Nicotine administration increased the mortality and severe lung damage by ~45% by increasing TNF-α, IL-6, cytokine-induced neutrophil chemoattractant (CINC)-3, and thrombin–antithrombin complexes, with slightly increased anti-inflammatory factor IL-10. Maybe the rat damage induced here was too severe to restore. After all, the anti-inflammatory effects of nicotine seem to affect the early stages of inflammation. Further studies are needed to better clarify the various effects of nicotine in established sepsis.

### 3.7 Acute Pancreatitis

#### 3.7.1 Anti-Inflammatory Effect of Nicotine on Acute Pancreatitis

Severe acute pancreatitis presents as a mild self-limiting disorder or a severe disease characterized by systemic inflammation and multiorgan failure, with a high mortality rate of 30%. Currently, no specific therapy is known.

Nicotine potentially mediated the development of pancreatic disease; not only prophylactic but also therapeutic application was effective in acute experimental pancreatitis using a glycodeoxycholic acid model of male Wistar rats. Nicotine (25 µg/kg per hour using an intravenous pump) reduced the pancreatic MPO levels and late cytokine release of serum HMGB1 levels but not TNFα, IL-1A, or IL-10; it significantly reduced systemic pancreatic inflammation and local necrosis (129).

Modulating the immunoregulation of CD4^+^CD25^+^ Treg is a critical mechanism for the nicotinic anti-inflammatory pathway for severe acute pancreatitis. Nicotine upregulated the number and suppressive capacity of CD4^+^CD25^+^ Treg by inducing the expression of immunoregulatory molecules CTLA-4, Foxp3, and TGFβ1 in the male C57BL/6 mouse model of retrograde injection of 50 μl of 2% Na-taurocholate into the pancreatic duct. Under this mechanism, nicotine (50, 100, and 300 μg/kg, i.p.) protected the mice from lethal severe acute pancreatitis in a dose-dependent manner and ameliorated inflammation, edema, acinar cell vacuolization, necrosis, and inflammatory cell infiltration. In addition, nicotine inhibited the MDA and MPO activities in tissue injury, production of digestive enzyme amylase and lipase in plasma, and production of pro-inflammatory cytokines TNF-α and IL-1β (130). It may be feasible to use selective agonists as immunotherapy for sterile local and systemic inflammatory diseases such as severe acute pancreatitis.

### 3.8 Myocarditis

#### 3.8.1 Anti-Inflammatory Effect of Nicotine on Myocarditis

Myocarditis may present with various symptoms, ranging from mild dyspnea or chest pain that resolves without specific therapy to cardiogenic shock and sudden death. Generally, myocarditis is induced by common viral infections; less commonly, other pathogens, toxic or hypersensitivity drug reactions, giant cell myocarditis, or sarcoidosis have also been implicated (131). Currently, there is no specific treatment for myocarditis.

The cholinergic anti-inflammatory pathway effectively protects the myocardium from viral infections. Cardiomyocyte apoptosis is critical for the development of coxsackievirus B3-induced myocarditis, which may result in heart failure or even sudden death. Li Yue-Chun’s team extensively investigated coxsackievirus B3-infected animal or cell models. Earlier studies have shown that nicotine treatments (1.2 mg/kg, i.p.) significantly improved the survival rates by 35%, attenuated myocardial lesions, improved the left ventricular function impairment, and downregulated the expression of TNF-α and IL-6, with no correlation with NF-κB p65 expression, but depending on the increased phosphorylation of STAT3 upstream in BALB/C mice (132). Furthermore, to investigate the dose-related effects of nicotine, doses of 0.1, 0.2, or 0.4 mg/kg injected intraperitoneally three times per day for 7 or 14 consecutive days were further evaluated. Notably, the low-dose nicotine was not effective when administered for up to 7 days, indicating a dose-dependent effect of nicotine treatment. However, the other nicotine treatment group exhibited reduced myocardial expression of pro-inflammatory cytokines (IL-1β, IL-6, IL-17A, and TNF-α) (133, 134). Furthermore, the effects of nicotine are independent of the integrity of the vagal nerve. However, vagotomy may reduce the release of acetylcholine from vagus nerve endings, with a decrease in the binding of α7 nAChR, finally increasing pro-inflammatory cytokines to aggravate inflammation. The cholinergic stimulation with nicotine also played a peripheral anti-inflammatory role (135). Later, they found that nicotine also contributed to its anti-inflammatory pathway in viral myocarditis by altering Th cell differentiation. Nicotine treatment (1.2 mg/kg, i.p.) increased Th2 and Treg cell proportions, decreased the proportion of Th1 and Th17 cells in the spleen, reduced the levels of pro-inflammatory cytokines IL-1, IL-6, and TNF-α in the heart, and finally decreased the severity of myocardium lesions and cellular infiltration in viral myocarditis (136). A recent study showed that, in coxsackievirus B3-infected neonatal rat cardiomyocytes and BALB/C mice, in addition to α7 nAChR, α3β4 nAChR subunits were also essential for nicotine-mediated anti-apoptotic effects and attenuating coxsackievirus B3 replication to protect cardiomyocytes *in vitro* (1 μM) and *in vivo* (0.2 mg/kg/d, i.p.). α3β4 nAChR/PI3K/Akt-dependent survivin upregulation is the established novel pathway in survival rate increase and heart function enhancement by nicotine (137).

The activation of the cholinergic anti-inflammatory pathway with nicotine reduces inflammation in autoimmune myocarditis. In cardiac troponin I-induced female A/J mice model, nicotine (for 3 days at 12.5 mg/L, 3 days at 125 mg/L, 21 days at 12.5 mg/L, and 21 days at 125 mg/L in drinking water after the first immunization) reduced inflammatory infiltration and fibrosis by downregulating the production of IL-6 and TNF-α in splenocytes, the myocardial expression of inflammatory proteins MCP-1, IL-1β, RANTES, CCR1, CCR2, and CCR5, and the myocardial expression of MMP14, NPPB (natriuretic peptide precursor B), TIMP-1, and osteopontin, which are involved in heart failure. The pSTAT3 activation played a key mediating role (138).

### 3.9 Allergic Diseases

#### 3.9.1 Anti-Inflammatory Effect of Nicotine on Allergy

Allergic rhinitis is characterized by several nasal symptoms, including sneezing, rhinorrhea, and nasal congestion, following provocation with the specific allergen. In addition, allergic rhinitis patients are characterized by the submucosal accumulation of inflammatory cells, including eosinophils, neutrophils, and T cells, accompanied by nasal hyperresponsiveness. Interestingly, in ovalbumin-immunized nasal responses of female BALB/C mice, environmental tobacco smoke also significantly suppressed allergen-induced nasal inflammation by suppressing nasal hyperresponsiveness and eosinophil accumulation. In the corresponding cell model of ovalbumin-specific Th2, nicotine (10^-6^ and 10^-7^ M) diminished cell proliferation and IL-4 production in a concentration-dependent manner, indicating that nicotine, at least in part, played an important role (139). In primary nasal epithelial cells from 19 healthy subjects stimulated by LPS, 50 μM nicotine for 24 h decreased the IL-8 release (0.6-fold), indicating the anti-inflammatory properties of nicotine in nasal inflammation (140).

Food allergies have become prevalent in developed countries over the past few decades. However, no effective drug therapies are currently available. In a severe allergic diarrhea model of BALB/C mice sensitized with repeated oral ovalbumin, nicotine treatment (3.2 mg/kg, s.c.) suppressed mucosal mast cell hyperplasia, elevated MPO, and the upregulated mRNA expression of Th1 cytokines (IFN-γ) and Th2 cytokines (IL-4 and IL-5) in murine colon. However, nicotine did not affect the increased plasma IgE levels. Nicotine treatment significantly ameliorated food allergy, mainly by suppressing upregulated mucosal immune responses *via* α7 nAChRs on immune cells. Thus, mucosal immune responses closely communicate with cholinergic nerve fibers to regulate mast cells critically involved in the pathogenesis of food allergies in mice (141).

Allergic asthma is a significant global health problem and sometimes becomes a life-threatening illness in all age groups. It is an inflammatory lung disease characterized by the pronounced infiltration of eosinophils and T cells into the submucosal tissues of airways, mucous cell hypertrophy/hyperplasia, increased mucus production, airway hyperresponsiveness, airway remodeling, and elevated production of total and allergen-specific IgE (142). The association between smoking and asthma is highly controversial. However, nicotine might be a good candidate for treating allergic asthma. In female Brown Norway rats sensitized with ragweed or house dust mite several times, nicotine (1 mg/kg; mini-osmotic pumps) decreased the mucus content in bronchoalveolar lavage and inhibited the accumulation of eosinophils and other inflammatory cells in the lungs by dramatically suppressing eosinophil trafficking and Th2 cytokine/chemokine IL-4, IL-5, IL-13, and IL-25 release, eotaxin responses, LTC4 secretion, and allergen-specific IgE, not IgG (143). Mazloomi et al. (144, 145) constructed a BALB/C mice allergic asthma model by alum and ovalbumin. The mice receiving nicotine (1 and 10 mg/kg 3 times every other day, s.c.) exhibited decreased allergic lung inflammation severity and allergic response intensity in a dose-dependent manner by potentiating Treg cell proliferation and TGF-β and decreasing the eosinophil count, bronchoalveolar fluid, IgE, and IL-4.

### 3.10 Obesity and Adipose Tissue Inflammation

#### 3.10.1 Anti-Inflammatory Effect of Nicotine on Adipose Tissue Inflammation

Obesity is characterized by chronic low-grade inflammation, contributing to metabolic syndrome such as fatty liver. Smoking cessation, often associated with weight gain, has led to the gradual recognition of nicotine’s fat-burning effects. Nicotine could reduce body mass by increasing physical activity and stimulating adipose tissue triglyceride lipolysis and brown adipose tissue thermogenesis (146, 147). Notably, nicotine significantly suppressed adipose tissue inflammation and further improved fatty liver diseases in genetically obese and diet-induced obese mice (27, 148). In genetically obese (db/db) and diet-induced obese C57BL/6J (B6) mice, nicotine (400 μg/kg, i.p. t.i.d) suppressed obesity-induced inflammation by normalizing the elevated expression of F4/80 and pro-inflammatory cytokines, including TNF-α, IL-6, IL-1β, and iNOS, and reduced the expression of MCP1 in adipose tissue or TNF-α in serum (148). Nicotine reduced the mRNA expression of inflammatory markers, such as PPAR-γ, TNF-α, and IL-6 in the liver of male Sprague–Dawley rats fed a high-fat diet at a dose of 2 mg/kg, s.c. In addition, nicotine improved hepatic damage and liver steatosis by reducing ER stress (146). Nicotine feeding (120 mg/kg) in CD-1 IGS mice also inhibited adipose tissue inflammation development by suppressing the inflammatory cytokines, including F4/80, TNFα, CD86, MCP1, ARG1, and IL-10, although it was not significant (147). The anti-inflammatory action of α7 nAChR stimulated by nicotine has also been demonstrated in stearic acid (C18:0) or TNF-α-induced 3T3L1 adipocytes (149) and α7 nAChR^-/-^ [α7 knockout (α7KO)] mice (148). Notably, α7 nAChR inhibited the acylation stimulating protein-induced expression levels of cytokines, MCP-1, and keratinocyte-derived chemokine (KC) *via* p38 kinase phosphorylation and NF-κB activation.

Nonalcoholic steatohepatitis (NASH), a severe form of nonalcoholic fatty liver disease, was alleviated by nicotine due to its anti-inflammatory effect. Tang’s group has carried out more research in this field (150–153). In male C57BL/6J (B6) NASH model mice fed with high-fat and high-fructose diets, nicotine (400 μg/kg, i.p., or 5 mg/kg, feeding) could significantly reduce the serum levels of IL-6 and TNF-α, and the inflammation was inhibited by the ERK/NF-κB/iκB pathway, TLR-4, and α7 nAChR stimulation in isolated Kupffer and LPS-induced RAW264.7 cells. In another study on a male NASH Wistar rat model fed with a l-amino acid-defined diet, nicotine (12 mg/kg, osmotic mini-pumps) significantly inhibited the induced hepatic inflammation and the increased expression of inflammation-related genes by regulating the expression of TNF-α, CD68, IL-1β, IL-6, Bax, and Cas3 mRNA in the liver and hepatic CD68-positive cells. Notably, the hepatic branch of the vagus nerve-induced anti-inflammatory response is essential for nicotine-activated α7 nAChR in NASH (154).

### 3.11 Anti-Inflammatory Effect of Nicotine on Other Inflammatory Diseases

The diseases listed in this part have not been studied very much, and mostly, the anti-inflammatory effects of nicotine have been shown. However, it has guiding significance for future research.

Systemic lupus erythematosus is an autoimmune disease characterized by aberrant immune function, increased renal inflammation, and hypertension. The cholinergic anti-inflammatory pathway is impaired in systemic lupus erythematosus because of autonomic neuropathy. However, nicotine stimulation (2 mg/kg, mini-osmotic pumps) could lower the blood pressure and proteinuria in female NZBWF1/J mice with systemic lupus erythematosus, a novel and appropriate disease model to study the link between chronic inflammation and the pathogenesis of hypertension. Additionally, chronic nicotine exposure inhibited splenic TNF-α and renal cortical MCP-1 and increased medullary IL-10. Thus, nicotine also regulated chronic renal inflammation through the immune and nervous systems acting together (155).

Uveitis frequently leads to severe vision loss and blindness by disrupting the blood–ocular barrier and neighboring tissues associated with intraocular proliferation. In LPS-induced Wistar rats of uveitis models, nicotine (1 and 2 mg/kg, i.p.) significantly reduced the protein, but not mRNA, levels of IL-6, CINC-1, and MCP-1, in serum and the levels of IL-6, IL-1β, CINC-1, TNFα, and MCP-1 in aqueous humor through α7 nAChR, suggesting the potential clinical translation of nicotine and other cholinergic agonists in uveitis (156).

Cholinergic signaling also suppressed inflammation in skeletal muscles under toxicant exposure, injuries, and diseases. In male mdx dystrophic mice with necrotic fibers and dense inflammatory infiltrates, nicotine treatment (400 μg/kg, i.p.) downregulated the activity of pro-inflammatory mediators MMP9, NFκB, and TNFα through acetylcholine secretion and F4/80^+^nAChRα7^+^ macrophage activation in mdx skeletal muscles, reduced the inflammatory infiltrates, and increased myofiber regeneration without increasing collagen deposition or fibrosis (157). In addition to regulating muscle inflammation, nicotine stimulated acetylcholine and activated muscle function and cell survival. In muscles and C2C12 myotubes of male FVB/N mice injected with paraoxon and/or purified recombinant AChE-R, nicotine (400 μg/kg, i.p.) reduced pro-inflammatory transcripts such as IL-6, CXCL1 (KC), CCL2 (MCP-1), and the transcriptional activity of NF-ĸB and AP-1 and increased anti-apoptotic protein Mcl-1 levels through tristetraprolin, which suppressed inflammation by destabilizing the mRNAs of pro-inflammatory cytokines (158). HO-1 and its downstream STAT3 are necessary to induce tristetraprolin by nicotine in LPS-treated mouse RAW 264.7 macrophages. The HO-1/STAT3/tristetraprolin/TNFα signaling pathway provides new possibilities for treating muscular inflammatory diseases (159).

Infectious or immunologic inflammation are important contributors to fetal growth restriction. Nicotine suppressed placental inflammation to protect the fetus in studies by the Liu group (160–162). In LPS-induced pregnant Sprague–Dawley rats, nicotine pretreatment (1 mg/kg, s.c.) suppressed the release of IL-1, IL-2, IL-6, TNF-α, and IFN-γ, but not IL-10 and IL-17, in maternal blood or placental tissues as well as the infiltration of leukocytes into the placenta through the cholinergic anti-inflammatory pathway by increasing α7 nAChR protein expression, which increased fetal survival and restored pup weight. Nicotine also reversed LPS-induced low levels of placental VEGF and placental pathological damage, possibly as an alternative therapeutic strategy for placental inflammation.

## 4 Conclusion

Nicotine is a well-known parasympathomimetic alkaloid, typically detected at a high level in tobacco leaves. The experimental studies reviewed here have suggested that the effect of pure nicotine monomer is not detrimental as cigarette smoke. Unlike pure nicotine, tobacco smoke contains toxic and carcinogenic substances. Although cigarette smoking has been defined as a global public health threat responsible for several diseases that can be partially prevented by quitting, smokers are less prone to some chronic inflammatory diseases. As yet, the dose and duration of nicotine treatment for these conditions is unclear. The discrepancy between the effects of nicotine on many inflammatory diseases might be attributed to the different pathophysiological mechanisms in these different models, the determined tissue, the treatment duration, or animal species (**Supplementary Table S1**).

**Figure 2:**
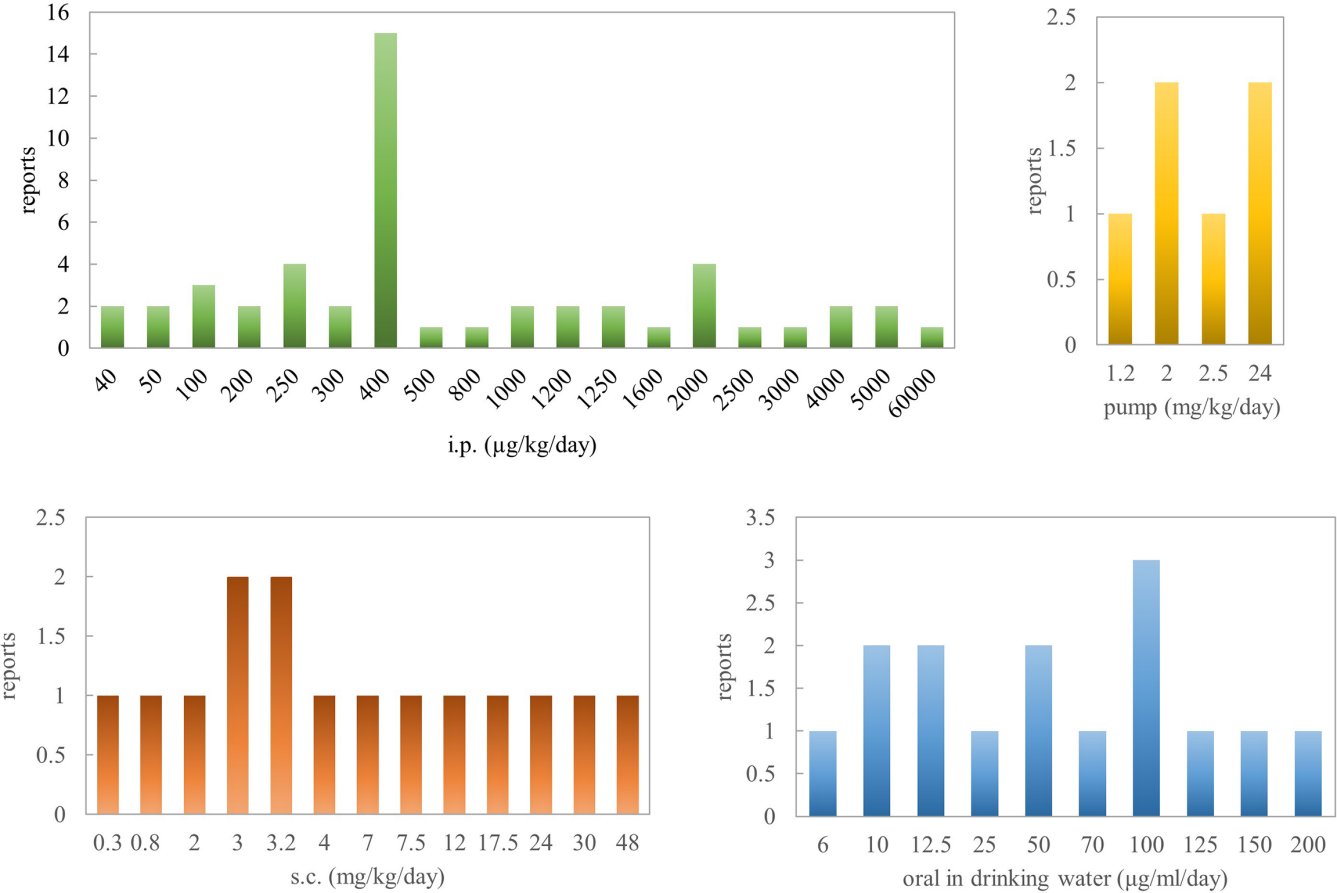
The dosage of nicotine salt used for *in vivo* animal experiments (i.p., intraperitoneal; s.c., subcutaneous).

Through the data analysis *in vivo* in mice (after uniform dose conversion in rats), the beneficial concentration range of nicotine in water is 6–200 μg/ml. The water intake of adult mice (~20 g) is generally 4–7 ml/day; after adjustment, the concentration is approximately 1–200 mg/kg. The concentration ranges of intraperitoneal injection, subcutaneous injection, and mini-pump infusion are 0.04–60, 0.3–48, and 1.2–24 mg/kg, respectively. After the conversion of effective utilization between different treatment methods, if all is injected intraperitoneally, the concentration range of nicotine is 0.04–600 mg/kg/day. Notably, the animal data on intraperitoneal injection are more comprehensive, and the effective dose is concentrated at 400 μg/kg/day (15 reports). A high dose is generally appropriate for acute and severe inflammation, such as sepsis (**Figure 2**). In fact, the concentration is calculated based on the nicotine ditartrate formula. The range of nicotine itself should be adjusted to 14 μg/kg/day to 210 mg/kg/day. It has also been suggested that nicotine may play a significant regulatory role in different diseases, especially acute and chronic inflammation as discussed here. Since there is not much data from animal studies, we did not find an approximate range of nicotine concentrations for pro-inflammatory effects. As the research progresses, it is also possible to use magic nicotine in clinical anti-inflammatory applications. In addition, we must consider an obvious problem because nicotine is closely associated with cancer progression, invasion, and metastasis (163). Therefore, nicotine’s application for anti-inflammatory purposes should be avoided in cancer patients. Since there is no evidence that nicotine itself provokes cancers, nicotine may be an alternative therapy for treating inflammation in non-cancer patients. The analyzed effective and safe dose range in this review may be meaningful to provide some references.

Of all the diseases summarized here concerning systemic inflammation, especially in sepsis and endotoxemia, nicotine exerted the most pharmaceutical effect and significantly improved the survival. Next, nicotine is also a potential candidate for treating ulcerative colitis, rheumatoid arthritis, osteoarthritis, multiple sclerosis, and myocarditis; the *in vivo* data provided a much better foundation. For local inflammation, the nicotine administration route may be more important to avoid its accumulation in other healthy organs—for example, the effect of nicotine on arthritis will be more pronounced when nicotine is directly injected into the focus of infection. Perhaps that is why, in the early years, tobacco was used to treat enteritis as enemas (4). It is evident that nicotine has a significant pro-inflammatory effect on periodontitis. However, the latest research also found that nicotine positively affects periodontitis at a lower dosage. In this regard, we consider that the effect of nicotine on periodontitis is mainly due to the influence of inevitable and original oral microbes. At present, most studies focus on the cellular level, and *in vivo* studies may be limited due to the difficulty of model construction. Therefore, we recommend that individuals with poor oral hygiene avoid excessive direct exposure to nicotine for oral diseases.

**Figure 3:**
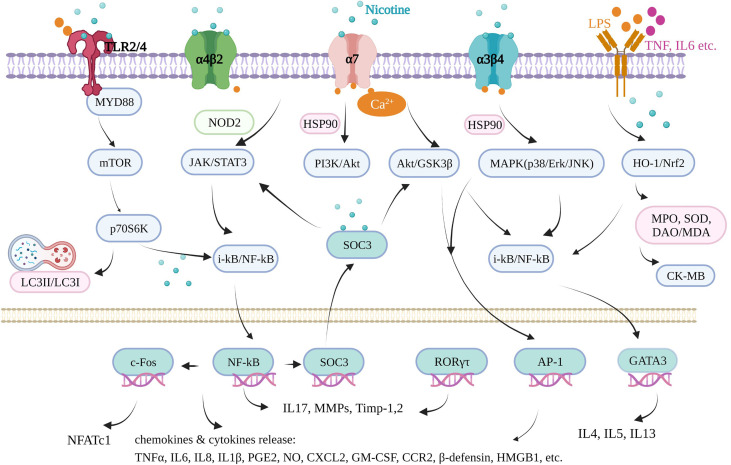
Schematic diagram of nicotine-mediated inflammation signaling pathways. Created with BioRender.

On the whole, nicotine can have an important anti-inflammatory function. The signaling network of nicotine’s regulation on inflammation is complex and involves several factors, including the vagus nerve, T cells, mononuclear phagocytes, and polymorphonuclear leukocytes, and neutrophil-mediated pro- (or anti-) inflammatory cytokine production through signaling pathways, such as STAT3/NF-κB, in many tissues. Nicotine not only acts on the vagus nerve but also on its hepatic or splenic branches to regulate acetylcholine receptors. Recent studies showed that, in addition to regulating the circulatory, respiratory, and digestive systems, the vagus nerve also regulates the immune system. On this basis, nicotine regulated immune cells, including CD25^+^Foxp3^+^ Tregs, CD4^+^IL-17^+^ Th17 Tregs, Th2 cells, CD4^+^CD25^+^ Tregs, F4/80^+^ macrophages, PD-1^-^IL-7R^+^CD8^+^ T cells, and so on, to balance the roles of immune cells. Furthermore, nicotine regulated the cytokine levels mediated by immune cells. To date, immune factors regulated by nicotine mainly include TNF-α, IL-6, IL-1β, PGE2, NO, LTB4, IFN-γ, IL-17, IL-8, IL-10, IL-12, IL-4, IL-1, IL-5, IL-13, IL-23, IL-25, IL-1α, CXCL2, GM-CSF cytokines, CCR2, and β-defensin. Nicotine plays a role in regulating inflammation mainly through the following pathways: (1) regulating oxidative stress balance through HO-1/MPO, SOD, and DAO/MDA, (2) regulating metal matrix enzyme pathways MMP1, MMP2, MMP3, MMP8, MMP9, MMP13, MMP14, and MMP28 and TIMP-1 and TIMP-2, and (3) regulating cell phosphorylation signaling pathways AMPK/mTOR/p70S6K/LC3II/LC3I, p62, beclin-1, IL-6/Stat3/miR-21, TLR2/MyDD88/IL-8, JAK2-STAT3-IL-6/MCP1, PI3K/Akt/iκB/NF-κB, p38/Erk/JNK/NF-κB, PI3K/MAPK (P38, JNK, ERK)/NF-κB, AP-1, NF-κB/c-Fos/NFATc1, HO-1/STAT3/tristetraprolin/TNFα, and HSP70/TNFα/iNOS. There are also specific target studies, including pro-inflammatory neuropeptide (substance P), occludin, ZO-1, RORγτ, GATA3, SOCS3, anti-cyclic citrullinated peptide (anti-CCP) antibodies, survivin, ECM molecules (collagen I, elastin, and fibronectin), PIN1, NEP, PU.1, cytokine-induced neutrophil chemoattractant (CINC)-3, and thrombin–antithrombin complexes, ARG1, NOD2, and VEGF. These factors form the signaling network of nicotine regulating inflammation (**Figure 3**).

Notably, many nicotine-induced effects in humans and animals are mediated through nicotinic acetylcholine receptors. nAChR α7 and other subtypes, such as nAChR α3, α4, α5, α6, α4β2, and α3β4, are generally activated by nicotine and expressed by numerous non-neuronal cell types, including distinct populations of astrocytes, epithelial cells, adipocytes, lymphocytes, macrophages, and keratinocytes, supporting the extensive anti-inflammatory effects of nicotine in many inflammatory diseases, even in the absence of parasympathetic innervation (100, 164). Among the nAChRs in these reviewed inflammatory diseases, it is not difficult to find that homomeric α7-containing nAChRs are the main ones with anti-inflammatory effects; heteromeric α4β2 and α3β4 also played mediating roles through regulating survivin or the apoptotic signaling pathway. Here α3, α4, and α6 subunits have no anti-inflammatory effects; pro-inflammatory effects were found only in oral diseases. In addition, it is noteworthy that the nAChR α7 subunit is the main regulator of macrophage/monocyte activation for the release of the molecules (TNF-α, IL-1β, and HMGB1) involved in inflammatory responses, reflecting the pro-inflammatory effect of nicotine in inflammatory diseases, especially oral inflammation.

The potential therapeutic use of nicotine is influenced by its complex pharmacology, diverse biological effects, adverse event profile, and the issues of tolerance, addiction, and safety. In particular, nAChRs have different effects under different conditions. Nicotine and partial agonists of nAChRs have the unique ability to regulate the network properties of groups of neurons and the immune system through the differential activation and desensitization of nAChRs on excitatory and inhibitory neuronal cell bodies and terminals (165). In inflammatory diseases, this is probably a similar-effect model as most other drugs concerning sensitivity or resistance. Therefore, future research about nicotine application and validation of the same disease is particularly important under different conditions, whether acute or chronic, short term or long term. However, an increasing understanding of the mechanisms by which nicotine interacts with the inflammatory system may soon open up further avenues for the therapeutic use of nicotine and other cholinergic agonists to combat several inflammatory disease processes. More importantly, further research is necessary to better balance anti-inflammatory and pro-inflammatory responses in inflammation-related and other diseases.

## Author Contributions

WenjiZ contributed to writing—original draft preparation. HL contributed to writing—reviewing and editing. MZ contributed to investigation. QY contributed to data curation. ZH contributed to investigation. XP and WenjuZ contributed to project administration and supervision. All authors contributed to the article and approved the submitted version.

## Funding

This work was supported by the National Natural Science Foundation of China (grant number 81903319), Natural Science Foundation of Guangdong Province of China (grant number 2021A1515011220), Administration of Traditional Chinese Medicine of Guangdong Province of China (grant number 20211008), Special Fund for Young Core Scientists of Agriculture Science (grant number R2019YJ-QG001), Special Fund for Scientific Innovation Strategy—Construction of High-Level Academy of Agriculture Science (grant number R2018YJ-YB3002), Top Young Talents of Guangdong Hundreds of Millions of Projects of China (grant number 87316004), the foundation of director of Crops Research Institute, Guangdong Academy of Agricultural Sciences (grant number 202205) and Outstanding Young Scholar of Double Hundred Talents of Jinan University of China.

## Conflict of Interest

The authors declare that the research was conducted in the absence of any commercial or financial relationships that could be construed as a potential conflict of interest.

## Publisher’s Note

All claims expressed in this article are solely those of the authors and do not necessarily represent those of their affiliated organizations, or those of the publisher, the editors and the reviewers. Any product that may be evaluated in this article, or claim that may be made by its manufacturer, is not guaranteed or endorsed by the publisher.
