# Supplementary material for: Nicotine in Inflammatory Diseases: Anti-Inflammatory and Pro-Inflammatory Effects
Source: Front Immunol. 2022 Feb 18;13:826889. doi: 10.3389/fimmu.2022.826889 (PMC8895249; doi:10.3389/fimmu.2022.826889)
Supplement: Supplementary file 1 [file Table_1.docx]

**Table S1 Effective dosage and targets of nicotine in inflammatory disease in different animal or cell models**

| **Diseases** | **Model** | **Nicotine dosage** | **Administration** | **Factors** | **Effect** | **Ref. (doi)/PMCID** |
| --- | --- | --- | --- | --- | --- | --- |
| **Inflammatory bowel disease** | | | | | | |
| Ulcerative colitis | dextran sodium sulfate (DSS) -induced male C57BL/6J mice | 0.1 mg/mL in drinking water | oral | MAdCAM‐1, | ++ | 10.1002/JLB.3A0717-304R |
| Ulcerative colitis | DSS-induced colitis of male C57BL/6 mice | 6, 12.5, 25, and 50 μg/ml in drinking water | oral | MPO, TNFα | ++ | 10.1124/jpet.112.198796 |
| Ulcerative colitis | DSS-induced colitis of male C57BL/6 mice | 0.1 mg/kg | s.c., t.i.d | MPO, TNFα | ++ | 10.1124/jpet.112.198796 |
| Ulcerative colitis | DSS-induced colitis of male C57BL/6 mice | 2.5 mg/kg/day | minipump infusion | MPO, TNFα | ++ | 10.1124/jpet.112.198796 |
| Ulcerative colitis | DSS-induced colitis of male C57BL/6 mice | 100 μg/ml in drinking water | oral | MPO, TNFα | -- | 10.1124/jpet.112.198796 |
| Ulcerative colitis | DSS-induced colitis of male C57BL/6 mice | 0.5 and 2 mg/kg | s.c., t.i.d | MPO, TNFα | -- | 10.1124/jpet.112.198796 |
| Ulcerative colitis | DSS-induced colitis of male C57BL/6 mice | 25 mg/kg/day | minipump infusion | MPO, TNFα | -- | 10.1124/jpet.112.198796 |
| Ulcerative colitis | DSS-induced colitis of male C57BL/6 mice | 10 µg/kg/day | gavage | TNFα, IL1β, IL6, AMPK, mTOR, P70S6K, LC3II/LC3I, p62, beclin-1 | ++ | 10.1016/j.cbi.2020.108943 |
| Ulcerative colitis | DSS-induced male C57BL/6J mice | 2 mg/kg | s.c. b.i.d | IL1β | ++ | 10.1152/ajpgi.00411.2011 |
| Ulcerative colitis | DSS-induced female C57BL/6 mice | 0.25 and 2.50 μmol/kg | i.p. | NF-κB, IL6, IL17, TNF, | + | 10.1111/j.1476-5381.2010.00699.x |
| Ulcerative colitis | 2,4,6-trinitrobenzene sulphonic acid (TNBS)- induced female C57BL/6 mice | 0.25 and 2.50 μmol/kg | i.p. | _ | - | 10.1111/j.1476-5381.2010.00699.x |
| Ulcerative colitis | DSS-induced male CD1 mice | 0.1, 0.3, 1.0 mg/kg | p.o. | α7 nAchR, IL1β | ++ | 10.1016/j.neuropharm.2012.06.004 |
| Ulcerative colitis | DSS-induced male BALB/c mice | 3 mg/kg | s.c. | MPO, α7 nAchR, IL6/Stat3/miR-21 | ++ | 10.1152/ajpgi.00346.2013 |
| Ulcerative colitis | a5 nAchR deficient mice | 12.5 mg/ml in drinking water | oral | PGE2, MPO, a5 nAchR | ++ | 10.1097/00001756-200507130-00018 |
| Ulcerative colitis | hapten 2,4-dinitrobenzene sulfonic acid (DNBS)-induced colitis in Sprague-Dawley rats | 8 mg/kg/day or 4 mg/kg | s.c., t.i.d | MPO, LTB4, MCP1, IL1β | ++ | 10.1093/toxsci/kfi238 |
| Ulcerative colitis | acetic acid-induced colitis of Sprague-Dawley rats | 1 mg/kg | i.p. | MDA, MPO, NF-κB, IL1β, visfatin | ++ | 10.1016/j.cbi.2013.06.009 |
| Ulcerative colitis | clostridium difficile toxin A-induced colitis in male Sprague-Dawley rats | 2 ng, 20 ng, 200 ng, 2 μg, 20 μg in 200 μl PBS | into the isolated colonic segments | TRPV1, LTB4, proinflammatory neuropeptide (substance P) | ++ | 10.1155/2016/4705065 |
| Ulcerative colitis | oxazolone- induced colitides of female BALB/c mice | 2.5 mg/kg | s.c. | CD25^+^Foxp3^+^ Tregs, CD4^+^IL17^+^ Tregs | ++ | 10.4049/jimmunol.1002711 |
| Ulcerative colitis | interleukin-10 deficient male C57/BL10 mice | 12.5 μg/ml in drinking water | oral | somatostatin and intestinal trefoil factor | ++ | 10.1097/00042737-200206000-00005 |
| Gut burn injury | male BALB/c mice | 400 μg/kg | i.p. | occludin, ZO-1, IFN-γ, TNFα, IL1β | ++ | 10.1016/j.ajpath.2012.04.005 |
| Small intestinal ulceration | indomethacin induced- male C57BL/6 mice | 0.3, 1, 3 mg/kg | i.p. t.i.d | MPO, iNOS, NFκB,  α7 nAChR | ++ | 10.1016/j.ejphar.2010.10.031 |
| Postoperative ileus | LPS-induced primary male Sprague-Dawley rats mesothelial cells | 10 nM | in vitro | IL1β, iNOS, α7 nAChR | + | 10.1292/jvms.17-0304 |
| Crohn's disease | trinitrobenzenesulphonic acid (TNBS) model of male Wistar rats model | 5 μg/mL, 100 μg/mL in drinking water | oral | MPO, iNOS, TNFα, LTB4 | ++ | 10.1007/s000110050597 |
| Crohn's disease | TNFα treated HT29 cells | 7.2 μg/ml | in vitro | nucleotide-binding oligomerization domain (NOD)2 (NOD2) | + | 10.1371/journal.pone.0024715 |
| Jejunum inflammation | interleukin-10 deficient male C57/BL10 mice | 12.5 μg/ml in drinking water | oral | somatostatin and intestinal trefoil factor | -- | 10.1097/00042737-200206000-00005 |
| Crohn's disease | trinitrobenzene sulfonic acid (TNBS)-induced BALB/c mice model | 2.5 mg/kg | s.c. | IL12 | -- | 10.4049/jimmunol.1002711 |
| Crohn's disease | mycobacterial (Mycobacterium avium paratuberculosis) -infected macrophages | 4 µg/mL | in vitro | TLR2/MyD88/IL8 | -- | 10.3390/microorganisms8111804 |
| **Arthritis** | | | | | | |
| Rheumatoid arthritis | collagen-induced arthritis (CIA) in male DBA/1 mice | 10, 100 μg/ml in drinking water (oral);  400 μg/kg( i.p.) | oral, i.p. | α7nAChR, TNFα, IL6 | ++ | 10.1002/art.24177 |
| Rheumatoid arthritis | CIA in male DBA/1 mice | 2 mg/kg in PBS | oral | IL17A, TNFα, IL6, CD4^+^IL17A^+^Th17 cells | ++ | 10.3109/14397595.2013.862352 |
| Rheumatoid arthritis | CIA in male DBA/1 mice | 250 µg/kg | i.p. | MIP‑1α, MCP‑1, CCR2 | ++ | 10.3892/mmr.2016.5904 |
| Rheumatoid arthritis | CIA in male DBA/1 mice | 250 µg/kg | i.p. | RORγτ, IL17A, IL4, GATA3 | ++ | 10.1016/j.ejphar.2014.04.019 |
| Rheumatoid arthritis | CIA in male DBA/1 mice | 250 µg/kg | i.p. | TNFα, IL6, HMGB1 | ++ | 10.1007/s10875-009-9346-0 |
| Rheumatoid arthritis | fibroblast-like synoviocytes isolated from rheumatoid arthritis patients | 0.1, 1, 10 µM | in vitro | IL6, IL8, NF-κB | + | 10.1007/s00296-010-1549-4 |
| Rheumatoid arthritis | TNFα stimulated fibroblast-like synoviocytes isolated from rheumatoid arthritis patients | 1-100mM | in vitro | IL6, IL8, NF-κB | - | 10.1007/s00296-010-1549-4 |
| Rheumatoid arthritis | TNFα stimulated fibroblast-like synoviocytes isolated from rheumatoid arthritis patients | 10 μM | in vitro | JAK2-STAT3-IL6/MCP1 | + | 10.1007/s10753-015-0117-1 |
| Rheumatoid arthritis | CIA in male DBA/1 mice | 250 μg/kg | i.p. | SOCS3, IL6 | ++ | 10.1080/03008207.2017.1380633 |
| Rheumatoid arthritis | TNFα induced fibroblast-like synoviocytes and LPS or IFN-γ treated monocytic cell lines (U937) | 0.1, 1, 10 μM | in vitro | MMP9, VEGF | + | 10.1080/08923973.2020.1745830 |
| Rheumatoid arthritis | killed Mycobacterium induced male Wistar rats | 2.5 mg/kg | oral | Rheumatoid factor (RF), C-Reactive Protein, NO, MPO, IL1, IL17 | ++ | 10.1016/j.lfs.2019.117037 |
| Rheumatoid arthritis | LPS stimulated whole blood from rheumatoid arthritis patients | 100 µM | in vitro | TNF | + | 10.1111/j.1365-2796.2010.02226.x |
| Osteoarthritis | monosodium iodoacetate injection in the C57BL/6J mouse model | 0.5, 1 mg/kg | i.p. | MMP9, PI3K/Akt-NF-κB, α7 nAChR | ++ | 10.4049/jimmunol.1801513 |
| Osteoarthritis | monosodium iodoacetate-induced osteoarthritis of male Sprague-Dawley rats | 1 mg/kg | i.p. | p38/Erk/JNK, NF-κB, α7 nAChRs | ++ | 10.1159/000369724 |
| Osteoarthritis | monoiodoacetate induced male Lewis rats | 0.625 mg/kg | i.p. | MMPs (MMP3,9,13), TIMP1, TNFα, IL6, IL1β | + | 10.1177/2050312116637529 |
| Osteoarthritis | primary chondrocytes of meniscectomy osteoarthritis of C57BL/6 mice model | 1, 10, 100 µM | in vitro | IL6, MMP3, MMP13, α7 nAChR | + | 10.1002/art.41429 |
| Osteoarthritis | male Sprague-Dawley rats model of early stage osteoarthritis by immobilizing the left knee joints | 50 μg/ml in drinking water | oral | TNFα, α7 nAChR | ++ | PMC4466929 |
| Rheumatoid arthritis | heat-killed M. tuberculosis H37Ra (Mtb)-induced arthritis of Lewis rats models | 0.625, 1.25, 2.5 mg/kg | i.p. | anti-cyclic citrullinated peptide (anti-CCP) antibodies, IL17, IL1β, TNFα, NO | ++(posttreatment) | 10.1002/art.30219 |
| Rheumatoid arthritis | heat-killed M. tuberculosis H37Ra (Mtb)-induced arthritis of Lewis rats models | 0.625, 1.25, 2.5 mg/kg | i.p. | anti-cyclic citrullinated peptide (anti-CCP) antibodies, IL17, IL1β, TNFα, NO | --(pretreatment) | 10.1002/art.30219 |
| Rheumatoid arthritis | chicken collagen II in complete Freud's adjuvant induced female ovariectomised Balb/c mice | 0.03% in drinking water | oral | survivin | -- | 10.1016/j.jaut.2016.12.009 |
| Osteoarthritis | interleukin-1β treated primary human articular chondrocytes from rheumatoid arthritis patients | 10^−8^ to 10^−7^ M | in vitro | MMP1, MMP13, fibronectin, chitinase 3-like protein 1 (CHI3L1) | -- | 10.1002/prca.201400186 |
| **Oral Disease** | | | | | | |
| Periodontitis | human oral epithelial cells HSC-2 line | 10^-8^-10^-3^ M |  | IL8, ICAM-1, β-defensin | + | 10.3390/ijerph18020483 |
| Periodontitis | male 5-week Wistar rats | 0.7 mg/kg | i.p. | TNFα, COX‑2 | -- | 10.3892/mmr.2017.7604 |
| Periodontitis | Fischer 344 rats | 0.8 mg/kg | injected the neck skin+intraperitoneally injected LPS | TNFα, TGFβ, IL10 | -- | 10.1111/j.1600-0765.2009.01223.x |
| Periodontitis | human periodontal ligament cells | nicotine (5 mM) and/or LPS (1 µg/mL) | in vitro | PKCa, Akt, GSK-3β, ERK, NF-κB, COX-2, TNFα, IL1β, IL6, IL17, RANKL | -- | 10.1111/jre.12332 |
| Periodontitis | human periodontal ligament cells | nicotine (5 mM) and/or LPS (1 µg/mL) | in vitro | PI3K/GSK3β/MAPK/ NF-κB, β-catenin, PGE2, NO, TNFα, IL1β, IL6 and IL12 and MMPs (MMP1, MMP2 and MMP9), ECM molecules (collagen I, elastin, fibronectin) | -- | 10.1111/jre.12240 |
| Periodontitis | human periodontal ligament cells | nicotine (5 mM) and/or LPS (1 µg/mL) | in vitro | PI3K/PKC/Akt/MAPK, NF-κB/c-Fos/NFATc1, iNOS, COX-2, NO, PGE2, TNFα, IL1β, IL8, TRAP(+) cells, phospholipase D (PLD) 1 and PLD2 | -- | 10.1902/jop.2015.150123 |
| Periodontitis | human periodontal ligament cells and/or the cocultures with CD4+ T cells | 10^-5^ M | in vitro | MMP1, MMP3, IL1β, IL6, IL17, IL21, CXCL12, a7 nAChR | -- | 10.1177/0960327115614383 |
| Periodontitis | human periodontal ligament cells | nicotine (10 mM) and LPS (1 µg/mL) | in vitro | HIF-2α, NO, PGE2, iNOS, COX-2, IL1β, TNFα, IL1β, IL6, IL8, IL10, IL11, IL17, MMPS (MMP1, -8, -13, -2 and -9), TIMPs (TIMP-1, -2), Akt, JAK2, STAT3, MAPK, NFκB, c-Jun, c-Fos | -- | 10.1002/jcb.25078 |
| Periodontitis | human periodontal ligament cells from periodontitis patients | nicotine (5 mM) and/or LPS (1 µg/mL) | in vitro | peptidyl-prolyl cis/ PIN1, NFκB, COX-2, iNOS, PGE2, NO, RANK, TNFα, IL1β, IL6, IL11, IL17, IL23 | -- | 10.1177/0022034514563335 |
| Periodontitis | human periodontal ligament cells | 5 mM nicotine- and 1 µg/mL LPS | in vitro | HO-1, NO, PGE2, iNOS, COX-2 | -- | 0.1111/j.1600-0765.2009.01215.x |
| Periodontitis | human periodontal ligament cells | nicotine (5 mM) and/or LPS (1 µg/mL) | in vitro | HO-1, iNOS, COX-2, NO, PGE2, IL1β, TNFα, IL6, IL12, PI3K/MAPK, PKC | -- | 10.1016/j.intimp.2009.08.015 |
| Periodontitis | human periodontal ligament cells | nicotine (5 mM) and/or LPS (1 µg/mL) | in vitro | HO-1, CORM-3, PGE2, COX-2, RANKL/OPG, CO | -- | 10.3892/ijmm.2017.3129 |
| Periodontitis | human periodontal ligament stem cells (PDLSCs) | 10^−9^ M nicotine, 5 ng/mL IL‐1β and/or 10 ng/mL TNF‐α | in vitro | ALP, RUNX2, BSP, OCN, RANKL/OPG, GSK‐3β, α7 nAChR | -- | 10.1111/jcmm.14986 |
| Gingivitis | gingival keratinocyte from healthy, non-tobacco-using subjects | nicotine (1 mM) + LPS (10 µg/mL) | in vitro | IL1α, IL8 | -- | 10.1111/j.1600-0765.2009.01262.x |
| Gingivitis | human gingival fibroblast (HGF) cell | 2.5, 10, 15 mM | in vitro | COX-2 | -- | 10.1034/j.1600-0765.2003.00681.x |
| Gingivitis | human gingival fibroblast (HGF) cell | nicotine (5 mM) and LPS (1 µg/mL) | in vitro | sirtuin 1, ROS, PGE2, TNFα, IL1β, IL6, IL8, IL17, PKC, PI3K, MAPK, NF-κB | -- | 10.1111/jre.12030 |
| Gingivitis | human gingival epithelial cells from subjects who had clinically healthy periodontium and no history of periodontitis | 0.3 and 1 mM | in vitro | β–defensin-2, IL8 | -- | 10.1111/j.1600-0765.2008.01153.x |
| Oral mucosal inflammation | human oral mucosal keratinocyte 100 (hOMK100) | 1 µM and 1 mM | in vitro | oral pain correlation factors substance P (SP), IL1β, neutral endopeptidase (NEP) | -- | 10.1111/eos.12072 |
| Oral mucosal inflammation | adult male Sprague-Dawley rats | Nicotine (EC50 557 μM) and capsaicin (100 μM) | in vitro superfusion of buccal mucosae | iCGRP, α3, α4 or α6 nAChR | -- | 10.1046/j.1460-9568.2003.02935.x |
| Oral mucosal inflammation | TNFα stimulated tissue and un-inflamed reconstituted human epithelium model | 10 mM | in vitro | IL1α, IL6, IL8, GM-CSF | no | PMID: 23472421 |
| Denture stomatitis | tryptic soy broth and denture biofilm | 4 mg/ml | in vitro | Streptococcus mutans, Candida albicans | -- | 10.1111/jopr.12643 |
| Pulpal inflammation | human dental pulp cells extracted from impacted third molars obtained from healthy patients | 5, 10, 50 μM | in vitro | MMP2, MMP28, MAPK | -- | 10.1016/j.lfs.2015.04.027 |
| **Skin Disease** | | | | | | |
| Ultraviolet radiation in the skin | female C57BL/6 mice back were exposed to ultraviolet radiation | The amount of nicotine in the drinking water is increased from 25 μg/ml on day 0 to 50 μg/ml on day 3, 100 μg/ml on day 5, and to the final concentration of 150 μg/ml on day 7. Mice are maintained on this concentration of nicotine for 6 weeks | oral | IL1β, α7nAChR, SOCS3 | ++ | 10.1016/j.jneuroim.2007.10.029 |
| Bechet’s disease | keratinocytes (isolated from normal neonatal foreskin specimens, which were removed during circumcision) and endothelial cells (HMEC-1) in the serum of patients | 6 μM | in vitro | IL8, IL6, HMEC-1, VEGF | ++ | 10.1038/sj.jid.5700492 |
| Passive skin arthus reaction | Wistar male rats | 0.4 mg/kg, s.c. 0.8 mg/kg, i.p. | s.c., i.p. | NO, TNFα | ++ | 10.1254/jphs.94.31; 10.1034/j.1600-0773.2003.920304.x |
| Skin wounds | full-thickness excisional skin wounds of C57BL/6 female mice and bone marrow derived macrophages, resident peritoneal macrophages and RAW 264.7 macrophages | 10^−8^ M and 10^−4^ M nicotine in 20 μl saline injected around the wounds. | injection, t.i.d | TNF, IL6, IL12,VEGF, PDGF, TGF-β1, TGF-β2, IL10 | -- | 10.1016/j.intimp.2013.10.022 |
| **Multiple sclerosis** | | | | | | |
| Multiple sclerosis | phytohemagglutinin stimulated peripheral blood mononuclear cells from multiple sclerosis patients | 10 μM | in vitro | IL1β, IL17, α7 nAChR | ++ | 10.1016/j.intimp.2015.06.034 |
| Multiple sclerosis | experimental autoimmune encephalomyelitis (MOG35-55 -induced female C57BL/6 mice) | 70 μg/mL nicotine in drinking water | oral | TNFα, IL6 | ++ | PMC3659034 |
| Multiple sclerosis | experimental autoimmune encephalomyelitis (MOG35-55 -induced female C57BL/6 mice) | 0.25 μL/h | mini-osmotic pumps | nestin, Ki67, M2 subtype microglia (NG2^+^ and CC1^+^) | ++ | 10.1016/j.neuroscience.2015.03.031 |
| Multiple sclerosis | in guinea pig spinal cord homogenate induced female Wistar rats with experimental autoimmune encephalomyelitis | 2.5 mg/kg | i.p. | IL10, IL17, TNFα, IFNγ | ++ | 10.1080/08820139.2017.1391841 |
| **Sepsis and endotoxemia** | | | | | | |
| Sepsis and endotoxemia | female C57BL/6 mice intraperitoneal injection with live Escherichia coli | 100 µg/mL in drink water | oral | TNFα, IL6, IL1β, AST, ALT | ++ | 10.1086/430323 |
| Sepsis and endotoxemia | LPS induced male Wistar rats endotoxemia model by tail vein injection | 0.1 mg/kg | i.v. | ALT, creatine kinase-MB (CK-MB) , TNFα, IL6, diamine oxidase (DAO), IL10 | ++ | 10.1097/TA.0b013e3181e9732d |
| Sepsis and endotoxemia | female C57BL/6 mice induced by cecal ligation and puncture | 400 µg/kg | i.p. | _ | ++ | 10.1097/01.CCM.0B013E31816208B3 |
| Sepsis and endotoxemia | LPS and cecal ligation puncture -induced male BALB/c mice | 40 µg/kg - 400 µg/kg | i.p. | NFκB, HMGB1 | ++ | 10.1038/nm1124 |
| Sepsis and endotoxemia | LPS and cecal ligation puncture -induced male BALB/c mice | 400 µg/kg | i.p. | TNFα, iNOS, HO-1, HMGB1, a7nAChR, PKCs, ROS, PI3K/Akt/Nrf2 | ++ | 10.1089/ars.2010.3555 |
| Sepsis and endotoxemia | LPS and cecal ligation puncture -induced male BALB/c mice | 50, 100, 200, 400 μg/kg | i.p. | TNFα, IL1β, IL6, TLR4, α7nAChR/PI3K, PU.1 | ++ | 10.1093/infdis/jit669 |
| Sepsis and endotoxemia | LPS-induced human macrophages | 100 nM, 1 μM, 10 μM | in vitro | IRAK-M,TNFα, JAK2, PI3K, STAT3 | + | 10.1371/journal.pone.0108397 |
| Sepsis and endotoxemia | cecal ligation puncture -induced male BALB/c mice | 400 μg/kg | i.p. | HMGB1 | ++ | 10.1084/jem.20052362 |
| Sepsis and endotoxemia | LPS induced endotoxemia model of male BALB/c mice | 2 mg/kg | i.p. | TNF, spleen macrophages in the red pulp and the marginal zone | ++ | 10.1073/pnas.0803237105 |
| Sepsis and endotoxemia | outbred albino mice of both genders intraperitoneal injection of 24-h culture of E. coli of sepsis | 17.5 ± 2 mg/kg, 7 ± 0.8 mg/kg | s.c. | TNFα, IL1β, IL6, MIP-2 | ++ | 10.1007/s10517-012-1803-8; 10.1007/s10517-012-1585-z |
| Sepsis and endotoxemia | Male Wistar albino rats of sepsis induced by ligation and puncture of the cecum | 30 mg/kg | i.p. | MPO, GSH, MDA | ++ | 10.1093/ntr/ntw198 |
| Sepsis and endotoxemia | LPS-evoked endotoxemia male Wistar rats | 25, 50, 100 μg/kg | i.v. | α7, α4β2 nAChRs, TNFα | ++ | 10.1016/j.ejphar.2018.07.008 |
| Sepsis and endotoxemia | LPS-evoked endotoxemia male Wistar rats | 2 mg/kg | i.p. | HSP70/TNFα/iNOS | ++ | 10.1016/j.pharep.2019.04.013 |
| Sepsis and endotoxemia | Sepsis model induced by cecal ligation and perforation in vesicle acetylcholine transporter knockdown (VAChTKD, C57BL/6J background) mutant mice | 400 µg/kg | i.p. | vesicular Ach transporter protein, TNFα, CXCL2 | ++ | 10.2174/1567202612666151026105915 |
| Sepsis and endotoxemia | cecal ligation and puncture -induced male Wistar rats | 400 µg/kg | i.p. | TNFα, IL6, cytokine-induced neutrophil chemoattractant (CINC)-3 and thrombin–antithrombin complexes (TATc), IL10 | -- | 10.1007/s10753-010-9204-5 |
| **Allergic disease** | | | | | | |
| Allergic rhinitis | ovalbumin-specific Th2 cell | 10^-6^, 10^-7^ M | in vitro | IL4 | ++ | 0.5415/apallergy.2020.10.e18 |
| Nasal inflammation | primary nasal epithelial cells | 50 μM | in vitro | IL8 | ++ | 10.1186/1471-2466-14-32 |
| Food allergy | severe allergic diarrhea BALB/c mice model which were sensitized with ovalbumin received repeated oral ovalbumin | 3.2 mg/kg | s.c. | MPO, IFN-γ, IL4, IL5, α7 nAChRs | ++ | 10.1371/journal.pone.0085888 |
| Allergic asthma | female Brown Norway rats, multiply sensitized i.p. with ragweed or house dust mite | 1 mg/kg | mini-osmotic pumps | IL4, IL5, IL13, IL25, LTC4, IgE | ++ | 10.4049/jimmunol.180.11.7655 |
| Allergic asthma | BALB/c mice allergic asthma model by alum and ovalbumin | 1, 10 mg/kg | s.c., t.i.d | TGF-β, bronchoalveolar fluid, IgE, and IL4 | ++ | 10.1016/j.intimp.2018.08.006;  10.4103/1735-5362.263555 |
| **Adipose tissue inflammation** | | | | | | |
| Obesity | obese (db/db) and diet-induced obese C57BL/6J (B6) mice | 400 μg/kg | i.p., t.i.d | F4/80, TNFα, IL6, IL1β, iNOS, MCP1 | ++ | 10.1210/en.2010-0855 |
| Obesity | male Sprague-Dawley rats fed a high-fat diet | 2 mg/kg | s.c., t.i.d | PPARγ, TNFα, IL6 | ++ | 10.1210/en.2013-1839 |
| Obesity | CD-1 IGS mice | 120 mg/kg | feeding | F4/80, TNFα, CD86, MCP1, ARG1, IL10 | + | 10.1016/j.fct.2018.01.058 |
| Obesity | stearic acid (C18:0) or TNFα induced 3T3‑L1 adipocytes,α7nAChR-/- [α7 knockout (α7KO)] mice | 1, 10 µM | in vitro | α7nAChR, acylation stimulating protein (ASP), MCP1, keratinocyte-derived chemokine (KC), NF-κB | + | 10.1210/en.2010-0855; 10.3892/mmr.2016.5630 |
| Non‑alcoholic steatohepatitis | male C57BL/6J (B6) NASH model mice fed with high-fat and high-fructose | 400 μg/kg, i.p.  5 mg/kg feeding | i.p.  feeding | IL6, TNFα, ERK/NF-κB/iκB, TLR-4, α7nAChR | ++ | 10.3760/cma.j.issn.1007-3418.2015.01.015 10.3760/cma.j.issn.1007-3418.2016.10.010 PMID: 27752155 10.3892/mmr.2015.4318 |
| Non‑alcoholic steatohepatitis | male NASH Wistar rats model fed with l-amino acid-defined diet | 12 mg/kg | osmotic minipumps | TNFα, CD68, IL1β, IL6, Bax, Cas3 | ++ | 10.1371/journal.pone.0180475 |
| **Acute Pancreatitis** | | | | | | |
| Acute Pancreatitis | glycodeoxycholic acid induced male Wistar rats | 25 µg/kg | intravenously pump | MPO, HMGB1 | ++ | 10.1097/MPA.0b013e3182a85c21 |
| Acute Pancreatitis | male C57BL/6 mice retrograde injection of 50 μL 2% Na-taurocholate into the pancreatic duct. | 50, 100, 300 μg/kg | i.p. | MDA, MPO, digestive enzyme amylase, lipase, TNFα, IL1β, CD4^+^CD25^+^ Treg, CTLA-4, Foxp3, TGFβ1 | ++ | 10.1097/MPA.0000000000000294 |
| **Myocarditis** | | | | | | |
| Myocarditis | coxsackievirus B3-infected Balb/c mice | 1.2 mg/kg | i.p. | TNFα, IL6, NF-κB, STAT3 | ++ | 10.1371/journal.pone.0112719 |
| Myocarditis | coxsackievirus B3-infected Balb/c mice | 0.1, 0.2, 0.4 mg/kg | i.p. | IL1β, IL6, IL17A, TNFα | ++ | 10.1038/srep15895; 10.1016/j.lfs.2016.02.003 |
| Myocarditis | coxsackievirus B3-infected Balb/c mice | 1.2 mg/kg | i.p. | Th1, Th17 cells, IL1, IL6, TNFα | ++ | 10.1080/21505594.2018.1482179 |
| Myocarditis | coxsackievirus B3-infected neonatal rat cardiomyocytes and BALB/c mice | in vivo (0.2 mg/kg, i.p.)  in vitro (1μM) | i.p. | α7 nAChR, α3β4 nAChR, PI3K/Akt, survivin | ++ | 10.1155/2019/9496419 |
| Myocarditis | cardiac troponin I -induced female A/J mice model | for 3 days at 12.5 mg/L; 3 days at 125 mg/L; 21 days at 12.5 mg/L; and 21 days at 125 mg/L in drinking water | oral | IL6, TNFα, MCP1, IL1β, RANTES, CCR1, CCR2, CCR5, MMP14, NPPB, TIMP-1, osteopontin, STAT3 | ++ | 10.1161/CIRCRESAHA.111.245563 |
| **Systemic lupus erythematosus** | | | | | | |
| Systemic lupus erythematosus | female systemic lupus erythematosus NZBWF1/J mice | 2 mg/kg | mini‐osmotic pumps | TNF‐α, MCP‐1, IL‐10 | ++ | 10.14814/phy2.13213 |
| Uveitis | | | | | | |
| Uveitis | LPS-induced Wistar rats of uveitis models | 1, 2 mg/kg | i.p. | IL6, CINC-1, MCP1, IL1β, TNFα, α7 nAChR | ++ | 10.1167/iovs.06-0644 |
| **Skeletal muscle inflammation** | | | | | | |
| Skeletal muscle inflammation | male mdx dystrophic mice with necrotic fibers and dense inﬂammatory infiltrates | 400 μg/kg | i.p. | MMP9, NFκB, TNFα, F4/80^+^nAChR α7^+^ | ++ | 10.1016/j.jneuroim.2010.06.005 |
| Skeletal muscle inflammation | male FVB/N mice were I.V. injected with 550 μg/kg Paraoxon, 1000 U purified recombinant AChE-R or a combination of both. | 400 μg/kg | i.p. | IL6, CXCL1 (KC), CCL2 (MCP1), NF-ĸB, AP-1, Mcl-1, tristetraprolin | ++ | 10.1016/j.bbamcr.2011.11.001 |
| Skeletal muscle inflammation | LPS-treated mouse RAW 264.7 macrophage cells | 1 mM | in vitro | HO-1/STAT3/tristetraprolin/TNFα | + | 10.1016/j.freeradbiomed.2013.09.027 |
| **Placental inflammation** | | | | | | |
| Placental inflammation | LPS (25 μg/kg, i.p.)-induced Pregnant SpragueeDawley rats | 1 mg/kg | s.c. | IL1, IL2, IL6, TNFα, IFN-γ, IL17, α7 nAChR, placental VEGF | ++ | 10.1016/j.placenta.2016.01.015; 10.1042/BSR20190386; 10.1016/j.ajog.2014.04.026 |

++: positive effect, anti-inflammation; +: less positive effect, anti-inflammation; --: negetive effect, pro-inflammation; -: less negetive effect, pro-inflammation; s.c.: subcutaneous; i.p.: intraperitoneal; p.o.: by mouth or orally; i.v.: intravenous; t.i.d: three times a day; b.i.d: twice daily
